# Antifungal activity of copper oxide nanoparticles derived from *Zizyphus spina* leaf extract against Fusarium root rot disease in tomato plants

**DOI:** 10.1186/s12951-023-02281-8

**Published:** 2024-01-12

**Authors:** Sozan E. El-Abeid, Mohamed A. Mosa, Mohamed A. M. El-Tabakh, Ahmed M. Saleh, Mohamed A. El-Khateeb, Maha S. A. Haridy

**Affiliations:** 1 Nanotechnology & Advanced Nano-Materials Laboratory (NANML), Plant Pathology Research Institute, Agricultural Research Center, Giza, 12619 Egypt; 2 Mycology and Disease Survey Research Department, Plant Pathology Research Institute, Agricultural Research Center, Giza, 12619 Egypt; 3 Zoology Department, Faculty of Science, Al-Azhar University, Cairo, Egypt; 4 Department of Pharmaceutical Chemistry, Faculty of Pharmacy, Horus University, Horus, 34518 Egypt; 5 Water Pollution Research Dep, National Research Centre, Dokki, Cairo, Egypt; 6 Central Lab of Organic Agriculture, Agricultural Research Center (ARC), 9 Gamaa St, Giza, 12619 Egypt

**Keywords:** Copper oxide, *Fusarium solani*, *Zizyphus spina*, Tomatoes, Enzymatic activity, Pollen grains

## Abstract

Incorporating green chemistry concepts into nanotechnology is an important focus area in nanoscience. The demand for green metal oxide nanoparticle production has grown in recent years. The beneficial effects of using nanoparticles in agriculture have already been established. Here, we highlight some potential antifungal properties of *Zizyphus spina* leaf extract-derived copper oxide nanoparticles (CuO-Zs-NPs), produced with a spherical shape and defined a 13–30 nm particle size. Three different dosages of CuO-Zs-NPs were utilized and showed promising antifungal efficacy in vitro and in vivo against the selected fungal strain of *F. solani *causes tomato root rot disease, which was molecularly identified with accession number (OP824846). In vivo  results indicated that, for all CuO-Zs-NPs concentrations, a significant reduction in Fusarium root rot disease occurred between 72.0 to 88.6% compared to 80.5% disease severity in the infected control. Although treatments with either the chemical fungicide (Kocide 2000) showed a better disease reduction and incidence with (18.33% and 6.67%) values, respectively, than CuO-Zs-NPs at conc. 50 mg/l, however CuO-Zs-NPs at 250 mg/l conc. showed the highest disease reduction (9.17 ± 2.89%) and lowest disease incidence (4.17 ± 3.80%). On the other hand, CuO-Zs-NPs at varied values elevated the beneficial effects of tomato seedling vigor at the initial stages and plant growth development compared to either treatment with the commercial fungicide or Trichoderma Biocide. Additionally, CuO-Zs-NPs treatments introduced beneficial results for tomato seedling development, with a significant increase in chlorophyll pigments and enzymatic activity for CuO-Zs-NPs treatments. Additionally, treatment with low concentrations of CuO-Zs-NPs led to a rise in the number of mature pollen grains compared to the immature ones.  however the data showed that CuO-Zs-NPs have a unique antifungal mechanism against *F. solani*, they  subsequently imply that CuO-Zs-NPs might be a useful environmentally friendly controlling agent for the Fusarium root rot disease that affects tomato plants.

**Graphical Abstract:**

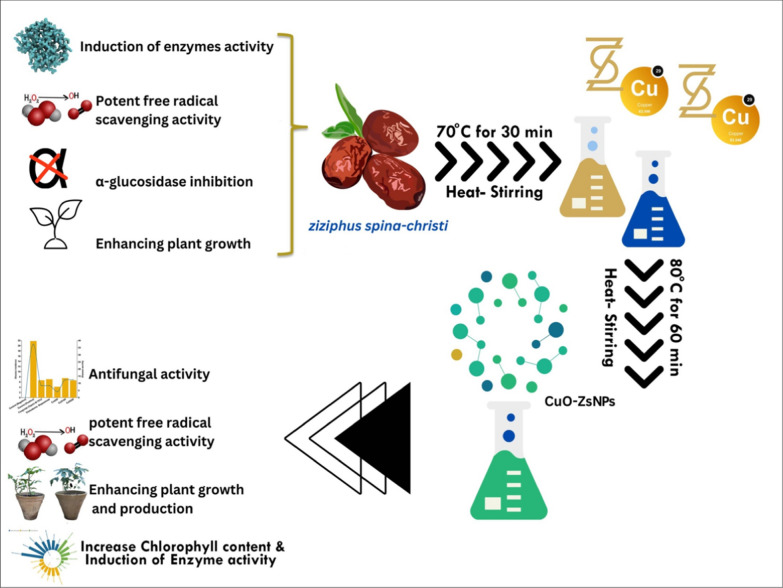

## Introduction

Tomato plants (*Lycopersicon esculentum* L.) hold significant importance as a vegetable crop, contributing to a global production of 130 million tonnes [71]. In Egypt, tomato cultivation covers approximately 32% of the total cultivated land, with tomato crops accounting for an estimated 16% of all vegetables grown [20]. The United Nations Food and Agriculture Organization (FAO) reported that Egypt had a growing area of 173.28 ha that produced 6.752 million tons yield of tomato fruits [24]. However, many fungal infections may infect tomato plants from the ground up in the form of seeds. These infections severely impact crop output, leading to considerable economic loss [96], Hamza, Mohamed, and Derbalah [32]; [59, 76]**.**

*Fusarium* is a notable fungal genus found in cropland soil, including various phytopathogenic species [58, 73]. Among these, the filamentous fungus *F. solani*, belonging to the *Fusarium solani* (FSSC) species complex [16] and *Nectria haematococca* is the species' sexual form. They also cause root rot in crops such as tomatoes, leading to severe yield loss [16, 75]. Although crop rotation has been documented as a safe method for controlling soil-borne diseases such as *Fusarium solani* (Liu, Yang, and Du [52]), it is often ineffective due to varying climate conditions and economic benefits. The traditional approach of seed dressing and field application with classical chemical fungicides have been utilized to reduce and suppress some *Fusarium* diseases [60]; However, the adverse impacts of this chemical fungicide on the environment and human health have pushed farmers to focus on creating harmless agricultural management practices. Therefore, there is an urgent need to identify a viable and harmless strategy for preventing the spread and development of *Fusarium* root rot.

Copper biocides have been widely implemented in the management of crop diseases due to their broad antimicrobial spectrum and low cost. However, bulk Cu fungicides are easily agglomerated, resulting in a decrease in their antimicrobial activity [40]. Another pathway to environmental contamination is the production and use of traditional copper-based fungicides, especially in the ionic form. According to Keller and Lazareva [44], a significant proportion of copper, approximately 15%, produced through manufacturing processes is discharged into soil, potentially leading to contamination of agricultural land. This poses a critical concern for food safety, particularly for extensively cultivated and consumed crops, such as tomatoes. To address this problem, different reports have indicated that Cu-based compounds and their oxides at the nanoscale are more stable and less able to interact with ecosystem elements than their bulk materials reflecting greater enhanced antimicrobial activity [95]. In addition, copper nanoparticles (CuNPs) have been reported to promote growth and nutrient accumulation in certain plants treated with them [46, 66]. As a result, researchers are suggesting environmentally friendly methods of synthesizing materials, with biomolecules being favored over other agents due to their protective properties [26]. The biosynthesis of copper nanoparticles using plant extracts as reducing and capping agents due to reduced properties in the leaf or fruit extract has emerged as an important branch of nanoparticle synthesis when conventional physical and chemical methods are too expensive, risky, time-consuming, and labor-intensive. However, the phytotoxicity of various metal nanoparticles, including silver, zinc oxide, titanium oxide, copper, and ferric NPs, on tomato plants has been evaluated in different studies. Thus, further research is needed to compare their toxicity with that of their bulk equivalents (Karami [3, 14, 57, 64, 80].

This study found that *Ziziphus spina-christi* (L.) wild leaves may effectively synthesize bioactive copper nanoparticles without negatively impacting the environment or the budget. Copper nanoparticles may be effectively reduced and stabilized using compounds in *Z. spina*-Christi leaves. This includes gallic acid, ellagic acid, hydrolyzable tannins, leucon anthocyanins, and flavanol glycosides (Cu-NPs). To our knowledge, no studies comparing the effects of nanocopper exposure to their bulk materials and Trichoderma-based biocides on soil-grown tomatoes have been undertaken. Therefore, the objectives of this study were to: (1) develop eco-friendly CuO NPs from *Zizyphus spina* leaf extract; (2) evaluate the antifungal properties of CuO-Zs-NPs against Fusarium root rot disease-causing agent under laboratory and greenhouse conditions; and (3) Ascertain the copper-based nanoparticle translocation capability and short-term exposure consequences in complex soil media. To accomplish this, tomatoes were grown for 120 days in soil amended with three different concentrations of CuO-Zs-NPs; (3) evaluate the impact of CuO-Zs-NPs on (seed germination, plant height, (fresh, dry) plant weight, and fruit weights,); and (4) quantification of chlorophyll pigment content and the enzymatic activity (polyphenol oxidase, peroxidase, hydrogen peroxide (H_2_O_2_) scavenging) linked to the production of reactive oxygen species (ROS), which was chosen as the end objective for determining the effects of nano-copper exposure. Additionally, this study aims to fill the Knowledge gaps regarding the mechanisms underlying the impact of CuO-Zs-NPs on the tomato plant life cycle, specifically concerning their impact on yield and seed fertility.

## Materials and methods

Analytical-grade chemicals were performed without any additional purification steps before use. Copper sulfate pentahydrate (CuSO_4_.5H_2_O, 98.0%), ammonia solution (NH_4_OH), and Potato dextrose agar were all purchased from (PDA)Sigma-Aldrich Co. A pathogenic isolate of *Fusarium solani* (OP824846) was originally isolated from tomato plants infected with root rot, grown in sterilized culture tubes, and reserved for further study at 4 °C on a PDA medium.

### Preparation of *Zizyphus spina* Christi plant extract:

*Zizyphus spina Christi* L (Zs) leaves were plucked from the Agricultural Research Center, garden, Egypt, and cleaned many times under running water to remove any remaining dust and dirt. Distilled water was used for flushing the leaves. In a 250 mL flask, 25 g of fresh leaves were combined with 100 mL of distilled water, and the resulting combination was heat-stirring at 70 °C for 30 min to produce an aqueous extract of the plant’s leaves. After allowing the extract to cool to ambient temperature, it was filtered using Whatman No. 1 filter paper until it was completely clear. Freshly used within one week, the extract was refrigerated and employed to produce copper nanoparticles.

### Synthesis of copperoxide nanoparticles

Approximately 10 mL of 25% aqueous extract from *Zizyphus spina* Christi was added gradually to 90 mL of 10 mM copper sulfate in a 250 ml Erlenmeyer flask to synthesize copper nanoparticles. For approximately 60 min, the mixture was heat-stirring continuously at 80 °C on a magnetic stirrer. CuO-ZsNPs were finally produced. This was preliminarily shown by the appearance of a dark green color, and the formed CuO-Zs-NPs solution was centrifuged for 30 min at 6000 rpm, and the clear supernatant was discarded. To purify CuO-Zs-NPs, the generated pellets were washed three times with distilled water using repeated centrifugation, dried in a vacuum oven at 60 °C for 24 h, after that calsinated at 300 °C for 1 h and then subjected to characterization with some microscopic and spectroscopic analyses.

### Characterization of copper oxide nanoparticles

The obtained CuO-Zs-NPs were confirmed through UV spectrophotometry (Thermo Spectronic, GENESYS-8, England, Quartz Cell, path length 10 mm). The hydrodynamic size distribution of the nanoparticles was determined using a Zeta Sizer Nano ZS Analyzer (Malvern Zeta Sizer Nano series, UK).

The morphology of the nanoparticles was observed by transmission electron microscopy (TEM, TECNAI 10Philips, Amsterdam, Netherlands).

Furthermore, to study the functional groups of CuO-ZS-NPs, the FT-IR spectrum was determined in the transmission mode (4000–400 cm^−1^) using a BOMEM FT-IR spectrometer (MB 147, Canada on KBr disk), a data library provider. A powder sample of approximately 100 mg was put in spectral grade KBr and pressed into discs under hydraulic pressure.

### Isolation and molecular identification of Fusarium isolate

During the summer of 2021, tomato plants exhibiting symptoms of Fusarium root rot were collected. Stem sections (3–5 cm in length) displaying root rot symptoms were thoroughly washed with tap water and then surface-disinfected for two minutes with a 2% chlorine solution. The small pieces were thoroughly rinsed with sterile double-distilled water (ddH_2_O) and then left to dry on filter paper under sterilized conditions before being placed into PDA medium (Potato + Dextrose + Agar) amended with 300 µg/ml streptomycin sulfate. Fourteen days were spent in a 25 ± 2 °C incubator with the fungal cultures. DNA analysis corroborated the fungal's phylogenetic position after they were identified following specified  mycokeys after a light microscope examination [35, 63]. The isolated *Fusarium* species was first cultivated on a PDA medium at 27 °C for 14 days to achieve molecular identification. The produced fungal mycelia were prepared as previously described [18]. The fungal DNA extraction was performed as stated by the steps of the DNeasy plant mini kit recommendation (Qiagen, Hilden, Germany).

### Molecular identification of *Fusarium solani*

The *F. solani* isolate was molecularly identified using the same PCR mixture and temperature conditions outlined to ensure its accuracy [18]. In addition, the ITS1 and ITS4 primers were used to amplify and sequence the ITS region of rDNA [98]. Two microliters of genomic DNA (10 ng/L), half a microliter of each primer pair (0.5 mM), and twelve and a half microliters of master mix (OmniPCR (MBA01-0100)) were used in a total volume of twenty-five microliters [15]. Initially, the amplification program consisted of a denaturation cycle for 3 min. at 94 ºC followed by 35 cycles (at 95 °C for 15 s, 53 °C for 30s. and 72 °C for 80 s). Finally, the extension step was 72 for 10 min. The obtained PCR product was visualized by electrophoresis on a 2% agarose gel. Using identical forward and reverse primers, the purified PCR products were sequenced. The experimental sequences for *F. solani *isolate have been deposited in the GenBank repository (NCBI).

### The activity of CuO-Zs-NPs against *Fusarium solani* in vitro

The produced Cu-Zs-NPs were tested for their antifungal activity against *F. solani* using the agar dilution method described in [59]. In this regard, 1mL of each concentration (50, 100, 250 mg/l) of CuO-Zs-NPs was supplemented separately in sterilized Petri plates containing 8 ml of sterilized melted PDA medium. The two solutions were mixed in a gentle circular motion to become homogenized before solidification. The commercial chemical fungicide “Kocide 2000, 2.5 μg/mL conc.” and Trichoderma Biocide* Trichoderma viride* 1.5% W.P. (One colony from a 7 days-old culture was inoculated in a 500 ml Erlenmeyer flask with 100 ml PDB to make biocide CFU count of 2 × 10^6^/gm minimum) served as controls to compare its activity with the produced CuO-Zs-NPs, while untreated plates were used as negative controls in this study. The flask was shaken at 200 rpm in an incubator at 27 °C for 7–10 days. To obtain cell-free supernatant, the Trichoderma culture was centrifuged for 15 min at 10000 rpm and filtered. Inoculating a Petri plate with 1 ml of cell-free supernatant, and then adding 8 ml of media). Thereafter, to evaluate the antifungal effect of CuO-ZS-NPs treatments on *F. solani*, a small disc (0.5 cm diameter) of active mycelial growth from the edge of an 8-day-old fungal culture was placed in the center of each prepared plate. The plates were then incubated at 26 °C ± 2 for 10 days. The radial growth of fungal hyphae was measured in all inoculated plates to determine the inhibition percentage using the formula:

$${\text{Inhibition }}\left( \% \right) \, = {\text{ T}} - {\text{t}}/{\text{T x 1}}00.$$

where(**T**) is the radial growth of fungal hyphae on the control plate and (**t**) is the radial growth of fungal hyphae in treatments [18]. All in vitro experiments were conducted in triplicate under sterile conditions to ensure the accuracy and reliability of the results.

### CuO-Zs-NPs phytotoxicity test

#### Germination assays

Tomato seeds were subjected to surface disinfection using a 2.5% NaOCl solution, followed by triple rinsing with distilled, sterilized water and air drying. To evaluate germination, 9 ml plates containing sterilized filter paper amended with various concentrations of CuO-Zs-NPs (50, 100, and 250 μg/mL), Kocide 2000 (a commercial fungicide) (Certis, Columbia, MD, USA), and Trichoderma Biocide were used under aseptic conditions at the recommended dose. Twenty-five seeds were placed on the surface of the sterilized filter paper in each plate (4 replicates/treatment) using sterilized forceps to ensure contact with the medium. The plates were incubated for six days at 25 °C ± 2 in a growth chamber with a 16 h:8 h day: night photoperiod. The experiment was repeated twice using twenty plates per treatment. The germination percentage (GP %) was calculated as follows: GP (%) = treatment an average of germination seed /control average of germination seed × 100.

### Greenhouse experiments

#### Preparation of soil medium

To investigate the impact of nano-copper exposure on tomato plants under sterile conditions, a soil medium consisting of loamy sand soil mixed with deionized water was prepared. The soil was amended with varying concentrations of CuO-Zs-NPs (50, 100, and 250 μg/mL), the chemical fungicide Kocide 2000 (Certis, Columbia, MD, USA), and Trichoderma Biocide. The pH of the soil was 7.9 [97]. The mixture was thoroughly mixed until homogeneity was achieved and allowed to settle for 24 h. Subsequently, four pots per treatment were filled with 0.5 L of the soil mixture, and three tomato seeds were planted in each pot. All pots were then transferred to environmentally controlled growth chambers for three weeks under a temperature cycle of 25/20 °C day/night, relative humidity of 65 –70%, and a photoperiod of 14 h with a light intensity of 340 μmol m^−2^ s^−1^.

### Effect of CuO-Zs-NPs on seed germination and plant growth

The efficacy of CuO-Zs-NPs on seed germination and some plant growth parameters of *Lycopersicum esculentus* L. plants was evaluated under greenhouse conditions. In this regard, ten seeds/replicates of tomato plants were first sterilized for 1–2 min in a solution of sodium hypochlorite (0.25% w/v). Seeds were then rinsed multiple times with ddH_2_O. Then, they were grown in small sterilized plastic bags filled with sterilized peat moss (0.5 kg) and treated individually with three different concentrations (50, 100, 250 μg mL^−1^) of CuO-Zs-NPs under greenhouse conditions. Additionally, treatment with both the chemical fungicide Kocide 2000 and Trichoderma Biocide was also used to compare their efficacy on seed germination and plant growth in comparison to CuO-Zs-NPs treatments and the nontreated tomato seed “control”. All treatments were evaluated in triplicate (3 pots in each replicate and 10 seeds/pot) at the 3–5 leaf stage, to study their efficacy on seed germination, seedling height, and seedling vigor index according to the following equation [50].

$${\mathbf{Vigor}} \, {\mathbf{index}} \, \left( {{\mathbf{VI}}} \right) \, = \, \left[ {{\mathbf{seedling}} \, {\mathbf{length}} \, \left( {{\mathbf{cm}}} \right) \, \times \, {\mathbf{seed}} \, {\mathbf{germination}} \, {\mathbf{rate}}} \right]$$

### Fungal inoculum preparation

To assess the effectiveness of CuO-Zs-NPs against Fusarium root rot, an inoculum suspension of *F. solani* was prepared using potato dextrose broth according to the method of Tesso et al., [83] with some modifications. The spore suspension was shaken and incubated at 160 rpm, and 30 °C for six days. After that the spore suspension was filtered and a sterile phosphate-buffered saline (PBS) solution was used to bring the spore concentration to the target value (1 X10^6^ conidia mL^−1^).

### In vivo experiments

#### Antifungal analysis of CuO-Zs-NPs against Fusarium root rot

The effectiveness of CuO-ZsNPs in combating Fusarium rot caused by *F. solani* was assessed in a greenhouse experiment using a susceptible strain of tomato (*cv*. TH99806). Three replicates of each treatment were conducted, with four to five seedlings in each replicate, using 25 cm diameter plastic pots filled with three kilograms of sandy, loamy soil mixed at a ratio of 1:1 (v:v) and autoclaved twice for thirty minutes at 121 °C. Each container was infected with 30 mL of a fungal spore solution containing 1 X10^6^ conidia mL^−1^ a week before the seedlings were transplanted. Tomato seedlings were subsequently exposed to varying concentrations of CuO-Zs-NPs (50, 100, and 250 g mL-^1^). Distilled water was used as the negative control, while Trichoderma Biocide and Kocide fungicide (2.5 g/L) were used as the positive controls. Then the treatments were repeated 30 days later. Every four weeks, 1 g L^−1^ of soluble NPK fertilizer was applied to the plants, and the plants were constantly examined for disease symptoms. After 45 days, growth data including plant length, biomass weight (Fresh + dry), as well as disease incidence (DI), and severity (DS) were recorded. Fresh biomass (g) was determined by removing excess soil from seedlings and their roots, whereas dry biomass (g) was determined by drying them in a vacuum oven at 60 ºC for three days. The severity of root rot disease was evaluated according to Romberg and Davis [72], using the following scale (0–4) with minor modifications. Disease severity rating scale (Showing brown taproots with slight to severe internal browning at the root tip): 0 = asymptomatic-healthy plants (none), 1 = less than 20%, 2 = 20–50%, 3 =  > 50–75% browning taproots (severe), and 4 =  > 75–100% leaves and roots with infection, total plant death, and drying.

The severity of the disease was determined using the following formula:

$${\mathbf{Severity}} \, \left( {{\mathbf{percentage}}} \right) \, = \, {\mathbf{PX}}\left[ {\left( {{\mathbf{n}} \, {\mathbf{V}}} \right)/{\mathbf{4}} \, {\mathbf{N}}} \right] \, {\mathbf{X100}}$$

**N** is the total number of plants tested for each treatment, **P** is the number of infected plants, **V** is the score of disease index, and 4 is the highest rate of disease index. Additionally, the following formula was used to determine disease incidence (DI):

$${\text{DI }} = \, \left( {{\text{Number of dead plants}}/{\text{Total number of plants}}} \right){\text{X 1}}00$$

Tomatoes were picked when mature on the 80–105th day following transplanting, at which point their uniformity and lack of damage could be verified, and the average fruit weight per plant was determined. On the 80–105th day after transplantation, tomatoes were harvested at the ripening stage, confirming that they were uniform and undamaged, and the average fruit weight per plant was also calculated.

### Physiological studies

#### Estimation of plant height, fresh and dry weight, fruit weight and Chlorophyll contents

In all treatments, the growth parameters and physiological effects of CuO-Zs-NPs on tomato plant height, fresh dry weight, fruit weight, and the level of certain photosynthetic pigments (chlorophyll a, b, and total chlorophyll content) were also assessed. For chlorophyll estimation, approximately 100 mg of leaf tissues from each set of treatments, including the mock-treated control, were weighed and extracted with 80% acetone in water (v/v) until the chlorophylls were completely released into the solution. Afterward, the suspensions were filtered, and absorbance at wavelengths 644 and 663 nm was measured. The concentration of photosynthetic pigments was evaluated based on Venkatachalam et al. [88]. Using the equations published by Lichtenthaler [51], chlorophyll a, b, and total chlorophyll were determined and expressed as mg/g-1 FW. Chlorophyll a = 12.25 × A663 − 2.79 × A645 chlorophyll b = 21.50 × A645 − 5.10 × A663.

### Estimation of enzymatic activities

#### Estimation of polyphenol oxidase (PPO)

To measure PPO activity, we used a technique originally developed by Kar and Mishra [39] with some modifications. We centrifuged 1 g of leaf tissue at 10,000 × g for 25 min at 4 °C after homogenizing it in 2 mL of 0.1 M sodium phosphate buffer (pH 6.5). The enzyme extract was prepared from the supernatant. The 3 mL of reaction mixture included 0.1 mM pyrogallol, 25 mM phosphate buffer (pH 6.8), and 0.1 mL enzyme extract. The reaction mixture was left without pyrogallol for calibration. The absorbance at 420 nm was recorded for each specimen.

#### The peroxidase (POD) activity

The approach presented by Gong et al. [29] was used to estimate the provided sample. Initial steps included grinding 1 g of leaf tissue in 5 ml of 0.05 M phosphate buffer (pH 7.0) containing 10% polyvinylpyrrolidone (PVP-SIGMA) and 0.1 M ethylene di-amine-tetra-acetic acid (EDTA-SIGMA). To utilize the supernatant for the POD test, the resultant mixture was centrifuged at 14,000 rpm for 20 min at 4 °C. The approach of Vetter et al. [89] was used to measure POD activity, with some adjustments made by Gorin and Heidema [30]. A 0.1 mM 2-morpholino ethane-sulfonic acid (MES) buffer, 0.05% hydrogen peroxide (H_2_O_2_), 0.10% phenylenediamine, and 0.10 ml (mL) of enzyme extract were used in the test combination (pH 5.5). Each sample's absorbance changes at 485 nm were recorded for 3 min.

### Hydrogen peroxide (H_2_O_2_) scavenging percentage

The percentage of hydrogen peroxide scavenging was determined from a methanolic leaf extract solution at a concentration of 1mg/10 ml and measured at 230nm wavelength according to the protocol of Eswaran, et al. [21]. In this regard, 1ml of the extract solution was gently mixed with 0.6ml (40mM) H_2_O_2_ prepared in phosphate buffer (pH 7.4). The final complete volume was 3 ml followed by incubation for 10 min at room temperature. Blank samples consisting of phosphate buffer without H_2_O_2_ were used in this experiment. The percentage of H_2_O_2_ scavenging in plant samples was calculated as indicated by Eswaran, et al. [21], following the equation:

$${\mathbf{H}}_{{\mathbf{2}}} {\mathbf{O}}_{{\mathbf{2}}} {\mathbf{scavenging}} \, \left( \% \right) = \, \left( {{\mathbf{A}}_{{\mathbf{0}}} - {\mathbf{A}}_{{\mathbf{1}}} } \right){\mathbf{X}} \, {\mathbf{100}}/{\mathbf{A}}_{{\mathbf{0}}}$$

where, A_0_ is the absorbance of the control samples, and A_1_ is the absorbance of the treated samples.

### Determination of Cu Content in Tomato terminal apical tissues

The concentration of the elemental Cu was determined, as described by Hernández-Hernández et al. [34]. First, tomato terminal apical tissues were harvested at maturation (120 days) and rinsed properly to remove the soil and dust particles, followed by extensive washing with distilled water and Air drying. The dried tissues were oven-dried at 50 °C for 72 h and gently ground to a fine powder. The digestion of 0.2 g of dry materials for analysis of Cu content was carried out by adding 30 ml of concentrated HNO_3_ to a 100 ml beaker. The beaker was covered with a watch glass and heated on the grill for approximately four hours until complete disintegration of the organic substance was achieved. The volume of HNO_3_ was adjusted periodically to prevent sample drying. Upon achieving a clear solution with no residue, the solution was cooled and filtered using Whatman No. 42 filter paper. The resulting filtrate was diluted with deionized water to a final volume of 50 ml in a volumetric flask. Subsequently, 1: 10 dilutions were prepared and analyzed for total Cu content using an inductively coupled plasma atomic emission spectrometer(ICP, ThermoJarrell Ash model 7400).

### Effect of Cu treatments on pollen grains

For the purpose of analyzing pollen grains, a number of flower bud samples were harvested from all treated and non-treated tomato plants. In this regard, flower buds of reliable size were randomly selected and fixed in a freshly made Carnoy's fixative solution (mixture of ethyl alcohol, chloroform, and glacial acetic acid in a volume ratio of 6:3:1). The anthers were then stained with 1% aceto carmine staining solution. The fertility of pollen was evaluated using stain-ability tests.

Staining of pollen grains was used as a proxy for fertileness; unstained or crushed pollen was considered sterile; and bursting pollen grains were also included in the count [19, 33].

Th**e** size of pollen grains that measured 1.5 times larger than the normally reduced pollen (n) in diameter was considered as unreduced (2n) pollen. Freshly visible pollen grains with clear characteristics were finally photographed using a HiROCAM (High-Resolution Optics Camera) digital imaging microscope eyepiece system [47].

### Statistical analysis

One-way analysis of variance (ANOVA) was performed on the collected data [7]. Using SPSS software version 22.0, means were compared using the Least Significant Difference (LSD) test (P < 0.05). The data shown here is the average of the three sets of results from each experiment, repeated three times (SD). The ability to visualize data are made available when utilizing MINITAB V.14 with R-studio V.4.1.3. GraphPad Prism 9 software was also used to make and edit graphs. The particle size was measured using Image J (version 1.53f), and Origin 2018 was used to create the particle size distribution histogram.

## Results and discussion

### Green synthesis of CuO-Zs-NPs

**Fig. 1:**
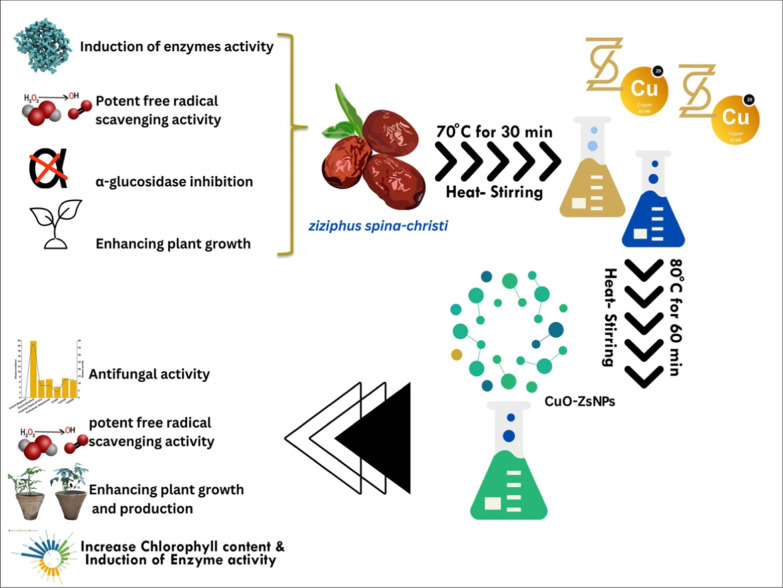
A schematic representation showing the possible mechanism for synthesizing the CuO-Zs- NPs

For the CuO-Zs-NPs to be formed, a solution of 90 mL copper sulfate was mixed with 10 mL of *Zizyphus spina* leaf extract at a ratio of 9:1 (v/v) and stirred continuously for 60 min at 80 °C. The color of the solution changed from blue to dark green during the reaction, indicating the formation of CuONPs. This was confirmed using UV‒vis spectroscopy, which showed the electronic spectrum caused by the surface plasmon resonance (SPR) effect [2, 5, 12, 28]. Previous studies have also reported the functional role of tannins, saponins, phenols, and alkaloids present in *Zizyphus spina* aqueous extract which was used as capping and stabilizing agents for green synthesis of CuO-Zs-NPs and their possible involvement in reducing Cu + to Cu0 [1, 45, 68, 93]. The mechanism for the synthesis is illustrated in Fig. 1.

### Characterization of the biosynthesized copper oxide nanoparticles(CuO-Zs-NPs)

#### UV Absorption of CuO-NPs

**Fig. 2:**
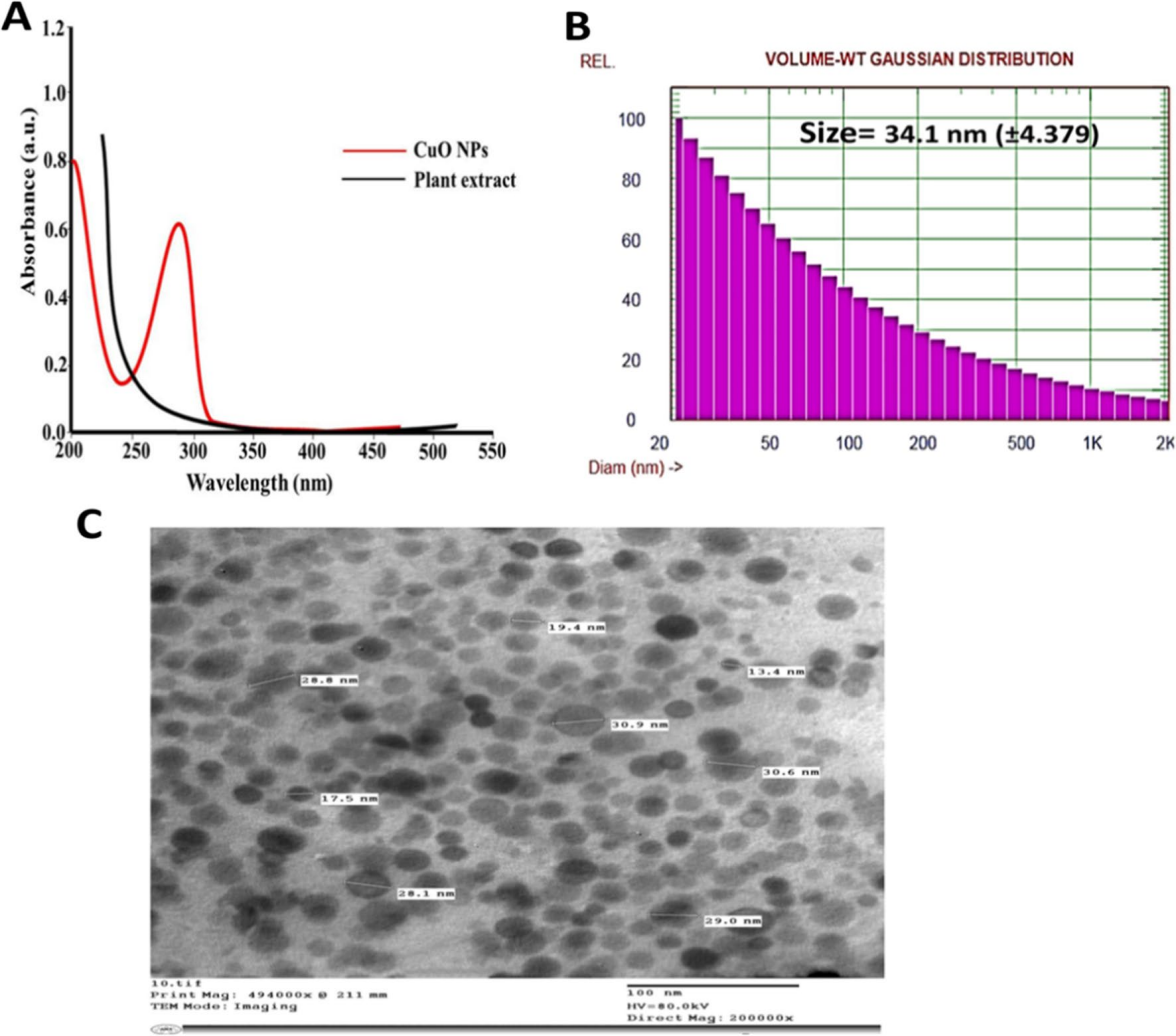
Characterization of the produced CuO-Zs-NPs: **A**: UV–vis spectroscopic analysis of the produced CuO-Zs-NPs and plant extract, **B**: Distribution analysis of CuO-Zs-NPs size, and **C**: Transmission electron microscope (TEM) image of the formed CuO-Zs-NPs

By analyzing the UV‒visible spectrum, a strong absorption peak located at approximately 290 nm was observed, which represents the successful synthesis of CuO nanoparticles (NPs) (Fig. 2A). The peak position and width are related to the average size and size distribution of the nanoparticles, respectively. This peak is attributed to the inter-band transition of core electrons in copper and the band gap difference associated with quantum size effects, which is consistent with the formation of various CuO NPs [27]. Furthermore, the absorption peak of excited CuO NPs that was noticed at 290 nm at a temperature of 23 °C, indicates a mono-dispersed size distribution in the mixture [69]. These results are compatible with those reported by Jing et al.(2019),which demonstrate the formation of stable CuO NPs [62].

### Morphology and size distribution of CuO-NPs

The morphology and size distribution of CuO-Zs-NPs nanoparticles (NPs) synthesized via a biosynthetic pathway were examined using Transmission Electron Microscopy (TEM), a crucial characterization technique for obtaining quantitative measures of particle size, size distribution, shape, and lattice fringes. The TEM image showed the appearance of spherical-shaped CuO-Zs-NPs with a particle size range of 13.4 − 30.9 nm (Fig. 2C). The size distribution measurements by dynamic light scattering were found to agree with the TEM analysis, yielding a particle size of mean at 34.1 ± 4.379 nm (Fig. 2B). However, slight variations in the two measurements can be due to many of variables. Dynamic light scattering (distribution analysis) investigates the hydrodynamic particle diameter in solution, which is based on the Brownian motion of particles in a solvent. Furthermore, temperature, viscosity, and particle translational diffusion coefficient all influence the hydrodynamic diameter in the solvent. The hydrodynamic diameter also takes account of all of the molecular sizes and hydration layers of water molecules.

### FT-IR analysis

**Fig. 3:**
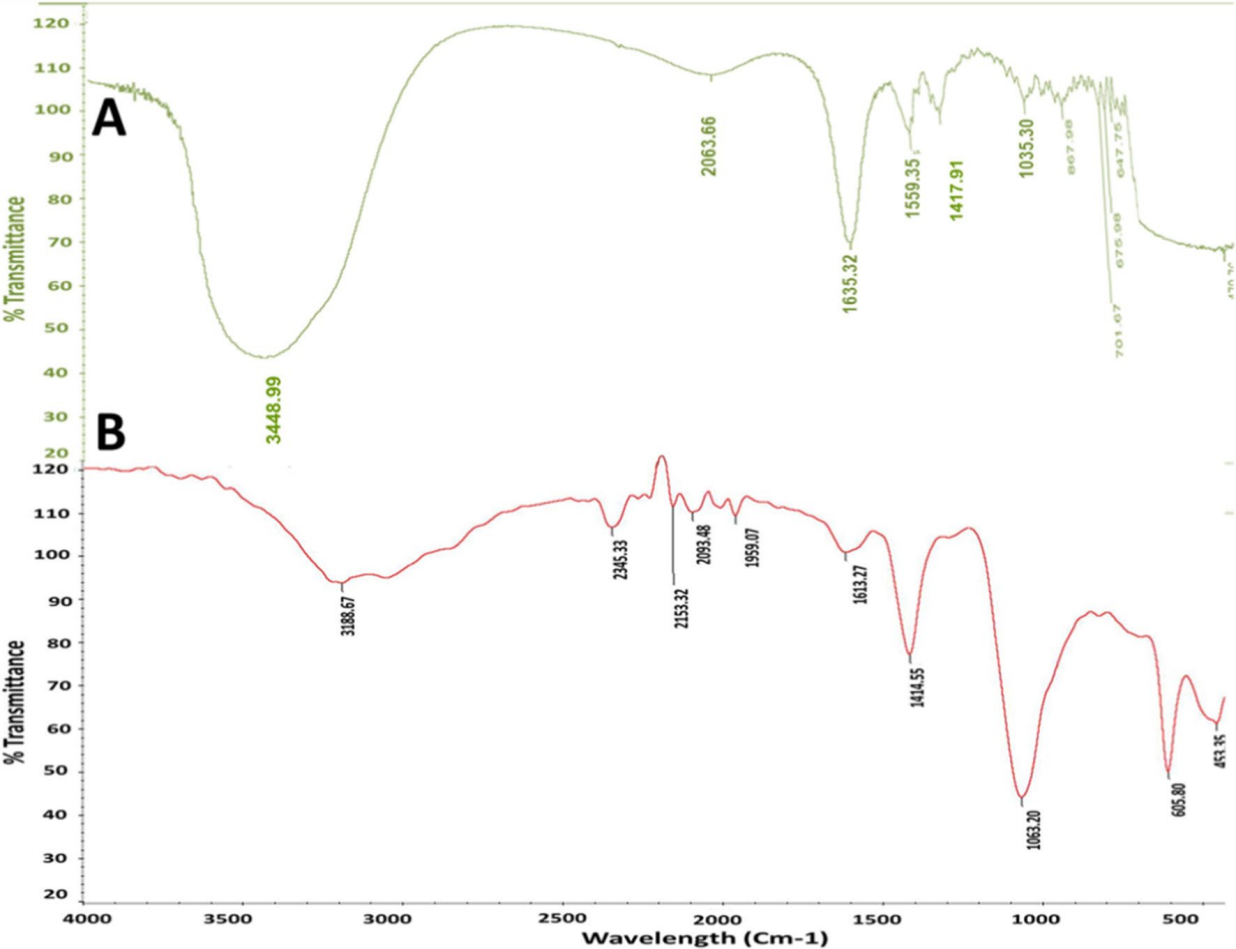
FT-IR spectrum analysis of *Zizyphus spina* leaf extract (**A**)  and the formed CuO-Zs-NPs (**B**)

To further examine the phytochemicals of *Zizyphus spina* leaf extract and their potential chemical functional groups responsible for the reduction and stabilizing of the CuO-Zs-NPs, FT-IR analysis was also performed on the produced CuO NPs to determine their chemical structure. As depicted in Fig. 3, the FT-IR spectrum shows absorption bands of *Zizyphus spina* leaf extract centered at 3448.99, 2063.66, 1635.32, 1559.35, 1417.91, 1035.30, 867.98, 701.67, 676.9, 647.76 and 470.7 cm^−1^(Fig. 3A). The broad absorption band at 3448.99 cm^−1^ may be attributed to the stretching vibrations of (–OH), while the peak at 2063.66 attributed to alene (C = C), and 1635.32 cm^−1^ may be attributed to the amine orenone group. While The peak at 1559.35 is due to the C = C stretching of the aromatic ring, in tertiary amides, flavonoids, terpenoids, tannins, and saponins. In addition, the peak at 1417 cm^−1^ could be due to C-N stretching in imines or oximes, or C-N stretching in aliphatic amines and 1035.30 are associated with C-O stretching in alcohols, phenols, ethers or esters also be due to C-N stretching in aromatic amines.

In Fig. 3B, the FT-IR absorption bands of CuO-Zs-NPs were centered at 3188.67, 2345.33, 2153, 2093, 1959, 1613, 1414,1063, 605, and 453 cm^−1^(Fig. 3B). The broad absorption band at 3188.67 cm^−1^may be attributed to the stretching vibrations of (–OH), while the peak at 2345.33 attributed to Carbon dioxide (O = C = O), and 2153cm^−1^ may be attributed to the presence of Si-based compounds. The peak at 1613 is due to N–H bending in primary amides, such as amino acids, and the C = C stretching in tertiary amides— flavonoids, terpenoids, tannins, and saponins. In addition, the peak at 1414 cm^−1^ could be due to C = N stretching in imines or oximes, or C-N stretching in aliphatic amines, and 1063 could be associated with C-O stretching in alcohols, phenols, ethers or esters also be due to C-N stretching in aromatic amines. Whereas the peak at 605 and 453 indicate the formation of O bonds, these data were attributed to [37].

### Molecular identification of *Fusarium solani*

Molecular identification of the causal agent of tomato root rot disease was performed using the internal transcribed spacer (ITS) region of ribosomal DNA. The ITS sequence of the isolated fungal strain showed 100% similarity with *F. solani* isolate sequence with the accession number (MW216959.1) available on the GenBank database. The identified ITS region of *F. solani* isolate was then assigned in the GenBank database with the accession number (OP824846).

### In vitro antifungal activity effect of CuO-Zs-NPs

**Fig. 4:**
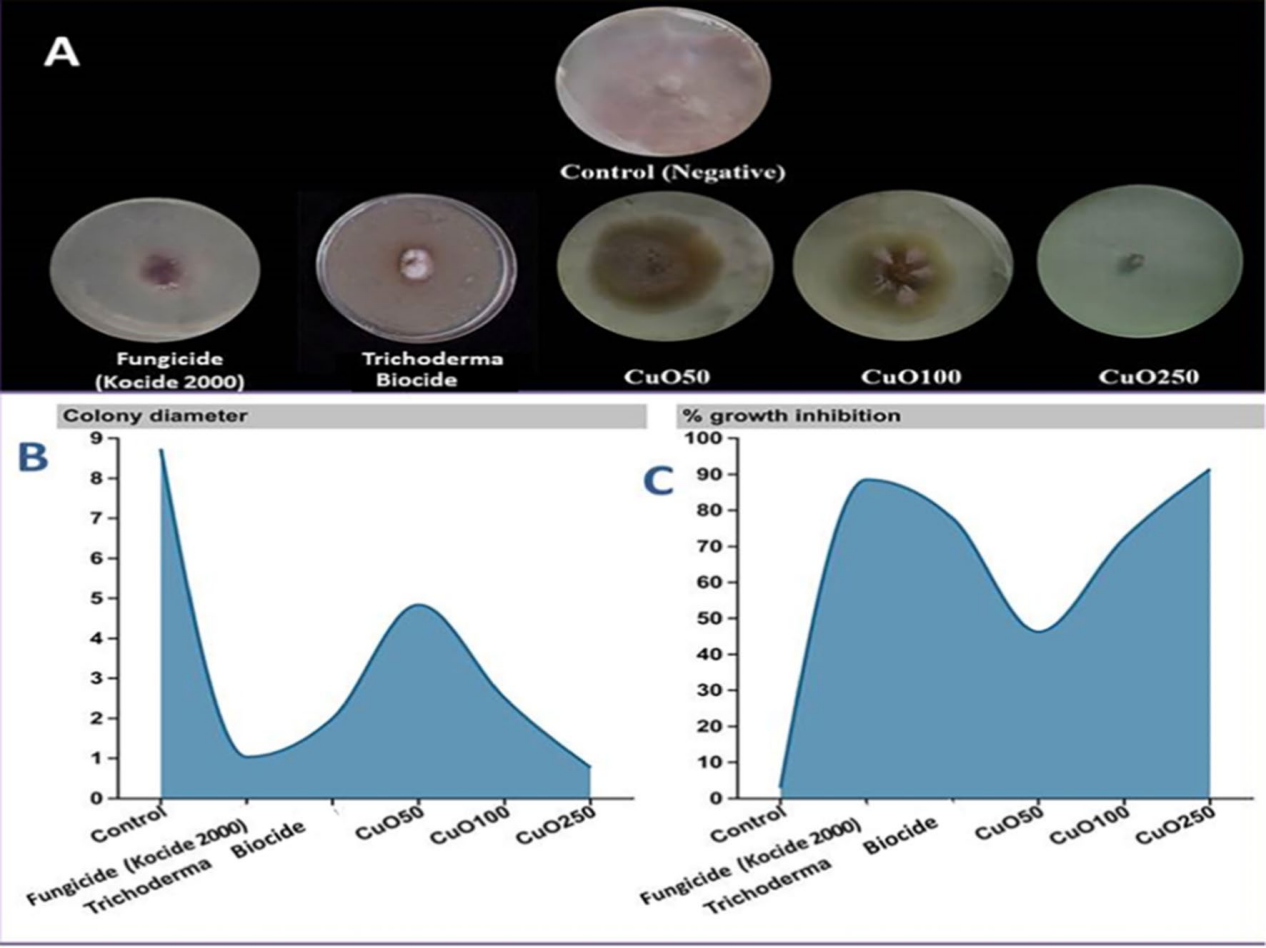
Antifungal activity of CuO-Zs-NPs at different concentrations as well as a commercial fungicide (Kocide 2000) and Trichoderma biocide against *Fusarium solani* on PDA medium **A**: Inhibitory activity of CuO-Zs-NPs against *F. solani* mycelial growth on PDA medium compared with Trichoderma biocide and the chemical fungicide, **B**: On colony diameter (cm) **C:** On percentage of growth Inhibition (%)

CuO-Zs-NPs anti-fungal activity was tested at various concentrations (50, 100, and 250 μg/mL), which are referred to in the manuscript as (CuO50, CuO100, and CuO250) respectively, in comparison with their bulk precursor of copper "Kocide 2000" as the chemical fungicide and TricodermaBiocide in terms of mycelial growth inhibition against isolated *F. solani *in vitro. The antifungal activity of CuO-Zs-NPs increased with increasing concentration (Fig. 4). Specifically, the highest levels of growth inhibition were achieved at a concentration of 250 mg/l for CuO-Zs-NPs. With a significant value of 91.17 ± 1.28, compared to Kocide 2000 as a chemical fungicide and the Trichoderma Biocide (*Trichoderma viride* 1.5% WP) showed values 88.20 ± 3.57 and 77.09 ± 5.88% after ten days of incubation (Table 1; Fig. 4). Interestingly, statistical analysis indicated that when examining the antifungal effect of the three tested CuO-Zs-NPs concentrations, the NPs concentration is a significant factor. Furthermore, this is in agreement with several studies showing that CuO-NPs display multiple inhibitory modes of action against microbial pathogens [31]. Additionally, Lopez-Lima et al. [53] tested Cu-NPs at different doses for *in-vitro *antifungal activity against *Fusarium oxysporum* f. sp. *lycopersici* (FOL). In addition, 0.5 mg/mL CuO-NPs inhibited mycelial FOL growth to 67.3%, compared to 15.6% for a commercial fungicide based on copper hydroxide, making them beneficial against various microbial pathogens that attack plants. Additionally, this would considerably help lower the risk of exposure to dangerous chemical fungicides, Furthermore, this would significantly contribute to reducing the hazardous effects of toxic chemical fungicides, especially on edible plants and fresh vegetables such as tomatoes [17, 41]. Taken together, our findings support that CuO-Zs-NPs are considered a promising replacement for traditional fungicides in crop protection.

**Table 1:** Effect of different concentration of CuO-Zs-NPs (50, 100, 250) mg/l, a chemical fungicide and Trichoderma biocide on the mycelial growth and Growth inhibition percentage (%) of *F. solani*

Treatments		Mycelial growth diameter (cm)	Growth inhibition (%)
CuO-Zs-NPs (mg/l)	CuO50	4.83 ± 0.29^b^	46.30 ± 3.21^a^
	CuO100	2.5 ± 0.5^c^	71.36 ± 5.56^b^
	CuO250	0.77 ± 0.12^d^	91.17 ± 1.28^c^
Trichoderma Biocide		2 ± 0.53^bc^	77.09 ± 5.88^b^
Chemical Fungicide (Kocide 2000)		1.03 ± 0.32^d^	88.20 ± 3.57^a^
Untreated Control		9 ± 0.0^a^	0 ± 0^d^

Letters on mean values statistically significant differences between the mean of the representive control and the treatments at the same time (p ≤ 0.01)

### Phytotoxicity test

#### Impact of CuO-Zs-NPs on the germination of tomato seeds

**Fig. 5:**
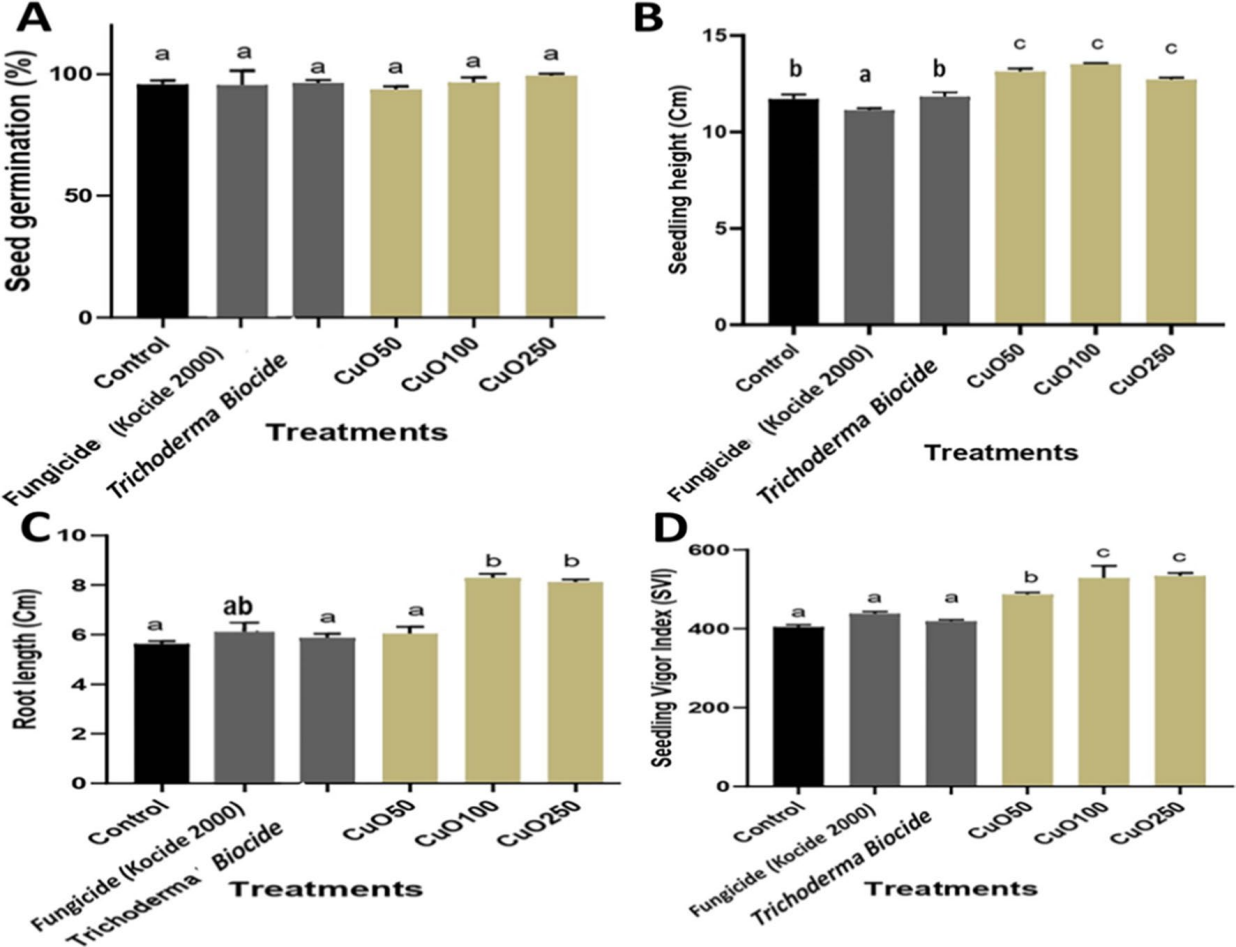
Effect of CuO-Zs-NPs 50, CuO-Zs-NPs 100, and CuO-Zs-NPs 250, the chemical fungicide “Kocide 2000” and Trichoderma biocide on **A** seed germination %, **B** seedling height, **C** Root length, and **D** seedling vigor index of tomato plants

The effects of different concentrations of CuO-Zs-NPs and its bulk counterpart (fungicide) in compared with Trichoderma biocide on seed germination and seedling length of tomato seeds were evaluated in vitro. The percentages of germination and germination index of tomato seeds treated with 50, 100, and 250 µg/ml CuO-Zs NPs compared to untreated control seeds are shown in Fig. 5**.**

The data presented in Fig. 5 indicates that all CuO-Zs-NPs treatments considerably had an inductive impact on seed germination ranging from 97.02 ± 1.53–98.67 ± 1.53 in compared to untreated control seeds giving values " 97.07 ± 1.54. Although treatments with the chemical fungicide and Trichoderma Biocide gave higher inductive values (98.33 ± 0.00, 98.0 ± 1.15) respectively for tomato seed germination, CuO-Zs-NPs at 250 mg/l showed the highest inductive impact on seed germination compared to other treatments The results also indicated that the addition of CuO-Zs-NPs particularly at 100 and 250 μg/mL did not negatively affect the number of germinated seeds compared to their bulk counterparts. Although treatment with Trichoderma Biocide showed a better effect on seed germination than treatment with CuO-Zs-NPs at 50 μg/ml.

#### Effect of CuO-Zs-NPs treatments on seed germination and growth vigor

**Fig. 6:**
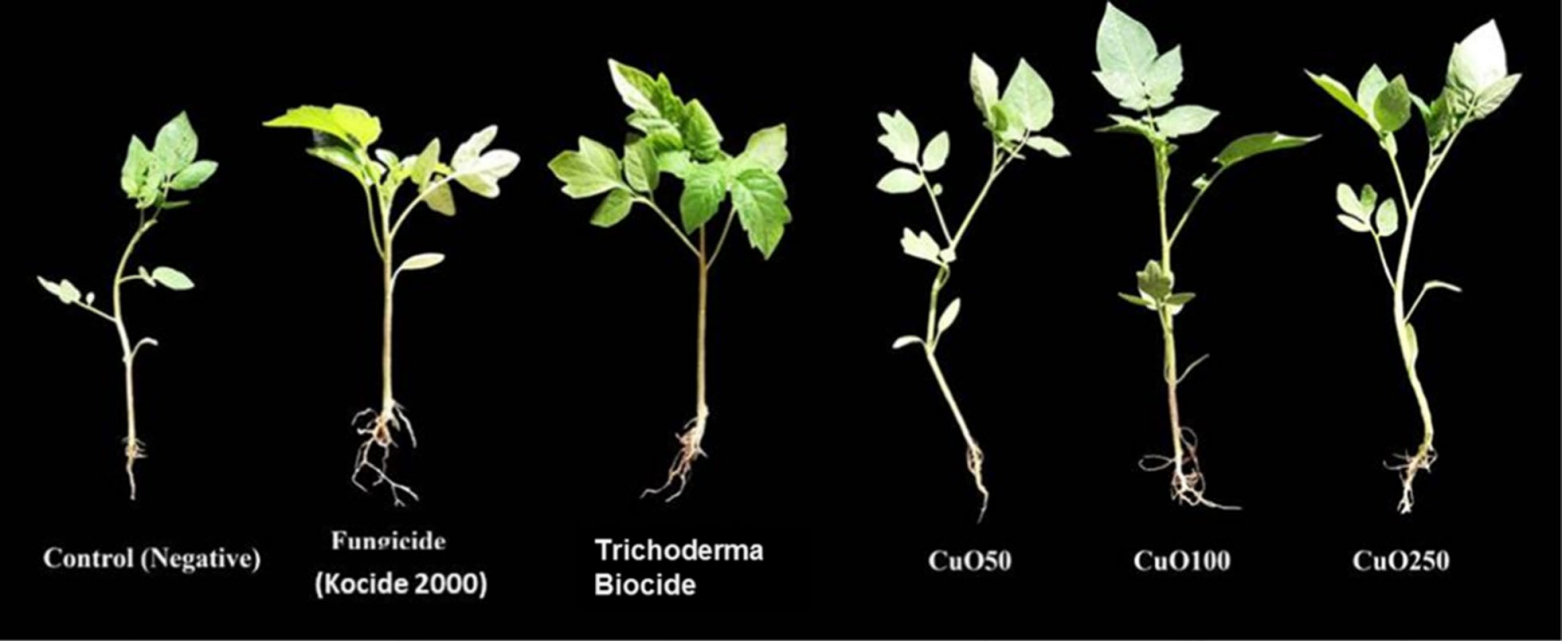
Effect of CuO-Zs-NPs 50, CuO-Zs-NPs 100, and CuO-Zs-NPs 250, the chemical fungicide "Kocide 2000" and Trichoderma biocide on tomato seedling growth

At the 3–5 leaf stage, the effect of CuO-Zs- NPs at concentrations (50, 100, and 250) mg/l on the percentage of tomato seed germination in soil medium was also evaluated and compared individually with the treatment of their counterpart (commercial chemical fungicide Kocide 2000) and Trichoderma Biocide (Fig. 5A). The results indicated that all treatments had no significant stimulating effect on seed germination compared to the untreated control. On the other hand, the results also indicate that the addition of CuO-Zs-NPs particularly at 100 and 50 μg/mL did not negatively affect the number of germinated seeds compared to their bulk counterparts. Similarly, the unique inductive effect of CuO-Zs-NPs particularly at 100 mg/l was also noticed in other tomato growth parameters such as seedling height, tomato root growth, and the vigor index (Figs. 5(B-D), and 6). A significant, dose-dependent impact on seedling height, root length, and Seedling vigor index was observed in all treatments with CuO-Zs-NPs compared to treatment with either Trichoderma biocide or the kocide fungicide (Fig. 5B–D). More interestingly treatments with CuO-Zs-NPs showed a more inductive effect on root length and seedling growth, in contrast to their bulk counterpart which showed a noticeable detrimental effect (Fig. 5). These data were confirmed by Pradhan et al. [66] tested Cu NPs on mung beans, and found that Cu-NPs stimulated plant growth better than copper sulfate. In contrast, Bakshi and Kumar [8] evaluated the effect of Cu-NPs on oregano, and reported that the negative or positive effects of nanoparticles depend on the plant type, nanoparticle concentration, and Cu oxidation state (Fig. 6).

#### Effectiveness of CuO-Zs-NPs against Fusarium root rot disease infecting tomato plants

**Fig. 7:**
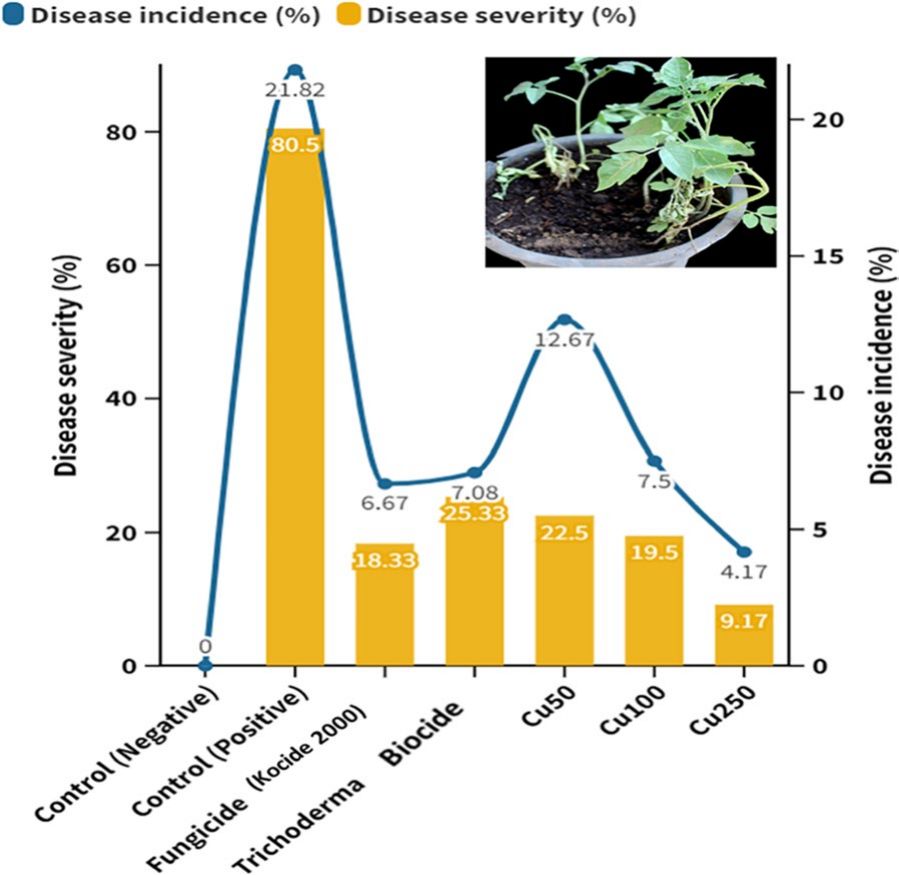
Disease incidence and severity of Fusarium root rot on infected tomato plants after treatment with CuO-Zs-NPs at (50, 100, 250) mg/L, chemical fungicide, and Trichoderma biocide compared to the positive and negative controls

The antifungal activity of CuO-Zs-NPs at three different concentrations (Cu50, Cu 100, Cu250) was further investigated in vivo experiment conducted to determine the efficacy of the treatment in controlling Fusarium root rot that affects tomato plants (Fig. 7). Disease severity was recorded for 90 days post inoculation (dpi) in all treated tomato plants. As a comparison, the experiment included plants treated with Kocide 2000 copper fungicide and Trichoderma biocide as chemical and biocide commercial controls respectively.

As early as 15 days post-inoculation, disease signs in mock-treated tomato plants were evident and the plants were visibly weakened; in contrast, disease symptoms in tomato-treated plants were minimal and greatly delayed. Control treatments of tomato plants either with an equivalent chemical fungicide, Kocide 2000, or Trichoderma Biocide also delayed and reduced disease symptoms, but to a lesser extent than CuO-Zs-NPs. A significant reduction in Fusarium root rot disease was noticed for all concentrations of CuO-Zs-NPs compared to the control treated with the pathogen only (infected control) (80.5%). No substantial difference was observed in the disease severity percentage in plants exposed to 50 or 100 mg/l CuO-Zs-NPs. Although treatments with either the chemical fungicide (Kocide fungicide) showed better disease reduction and disease incidence with values of 18.33% and 6.67% respectively, than CuO-Zs-NPs at a concentration of 50 mg/l, however, CuO-Zs-NPs at a dose of 250 mg/l achieved the highest disease reduction (9.17 ± 2.89%) and lowest disease incidence (4.17 ± 3.80%).

### Growth and Physiological parameters

#### Effect on plant height, weight, and chlorophyll content

**Fig. 8:**
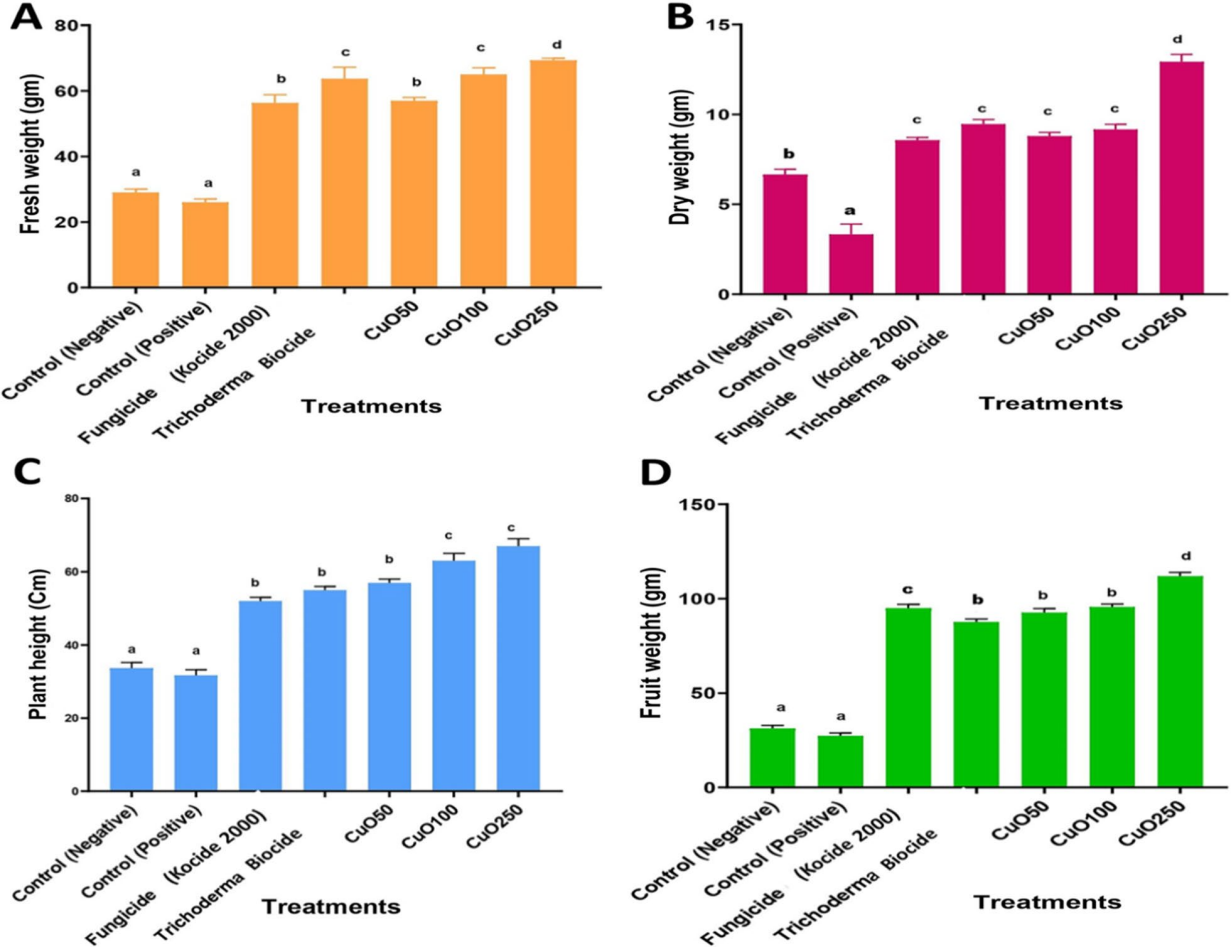
Effect of different concentrations of CuO-Zs-NPs (50, 100, 250 mg/L), the chemical fungicide "Kocide 2000" and Trichoderma biocide on **A** Fresh weight, **B** Dry weight, **C** Plant height, and **D** Fruit weight of tomato plants challenged with *F. solani*

**Fig. 9:**
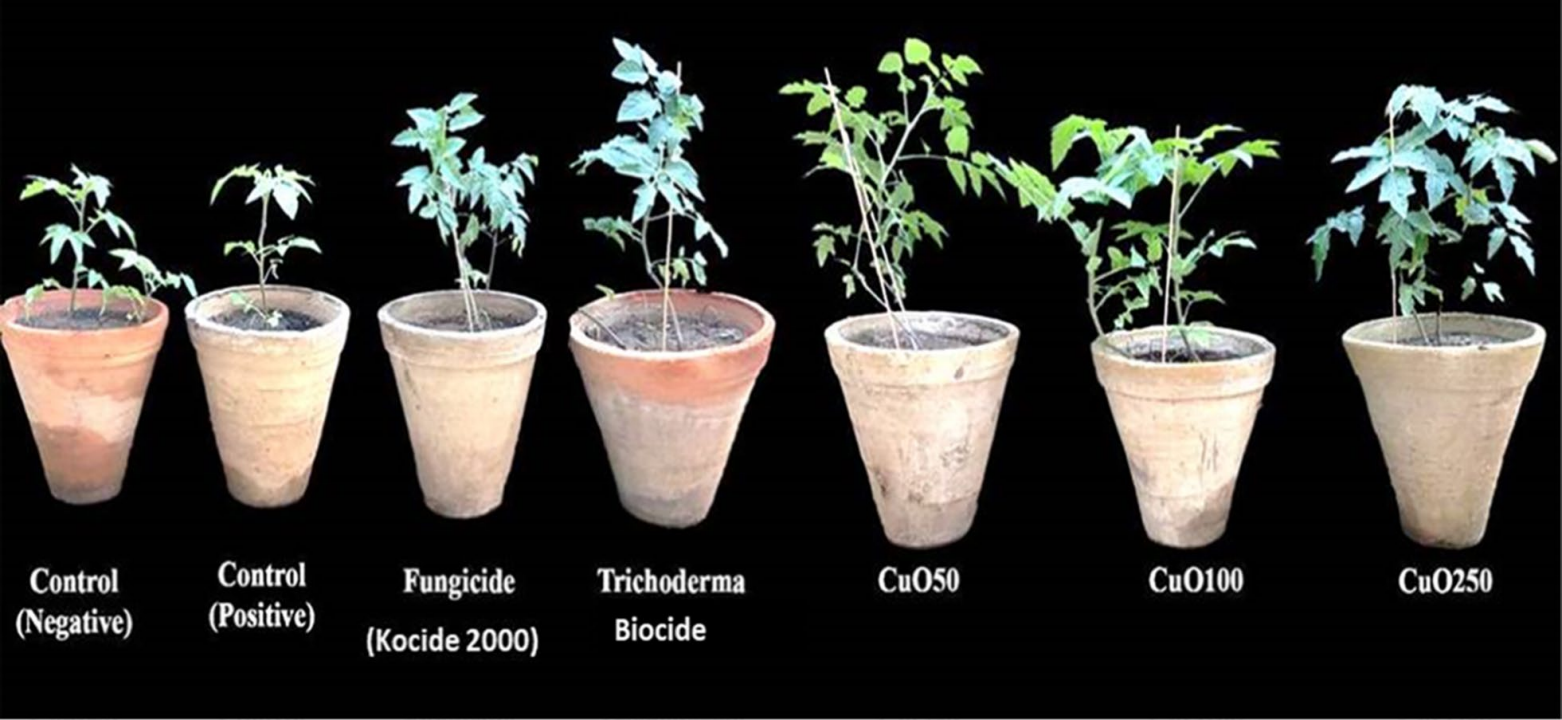
Effect of different concentrations of CuO-Zs-NPs (50, 100, 250 mg/L), the chemical fungicide "Kocide 2000" and Trichoderma biocide on tomato plants  grown in pot experiments treated with *F. solani* compared with the control groups

Three months after seeding, the effects of CuO-Zs-NPs on tomato seedlings were examined for plant lengths, fresh and dry weights, and weight of the resulting fruit (Fig. 8). The results indicated that all CuO-Zs-NPs had a promoting effect on the plant height and tomato (fresh, dry, fruit) weight. In this regard, CuO-Zs-NPs at (50, 100, and 250) mg/l concentration showed an inductive effect on tomato plant height with values 57.67 ± 12.5, 62.33 ± 7.37, and 65.33 ± 12.32 respectively, compared to 54.67 ± 3.05, 55.67 ± 3.51, and 37 ± 2.65 for treatment with Kocide 2000 fungicide, Trichoderma biocide, and the negative control respectively (Fig. 9). Similarly, the unique inductive effect of CuO-Zs-NPs, particularly at 250 mg/l, was also noticed in data reported for tomato fresh weight, dry weight, and fruit weight. Overall, our data indicate the effectiveness of Cu-Zs-NPs in reducing the disease severity and increasing all growth parameters and production, which was confirmed by Hernández-Hernández et al. [34]. In addition, the increase in dry weight may be a result of the NPs boosting the activity of photosystems I, II (PSI and PSII), along with the redox state of plastoquinone in the electron transport chain [79].

**Fig. 10:**
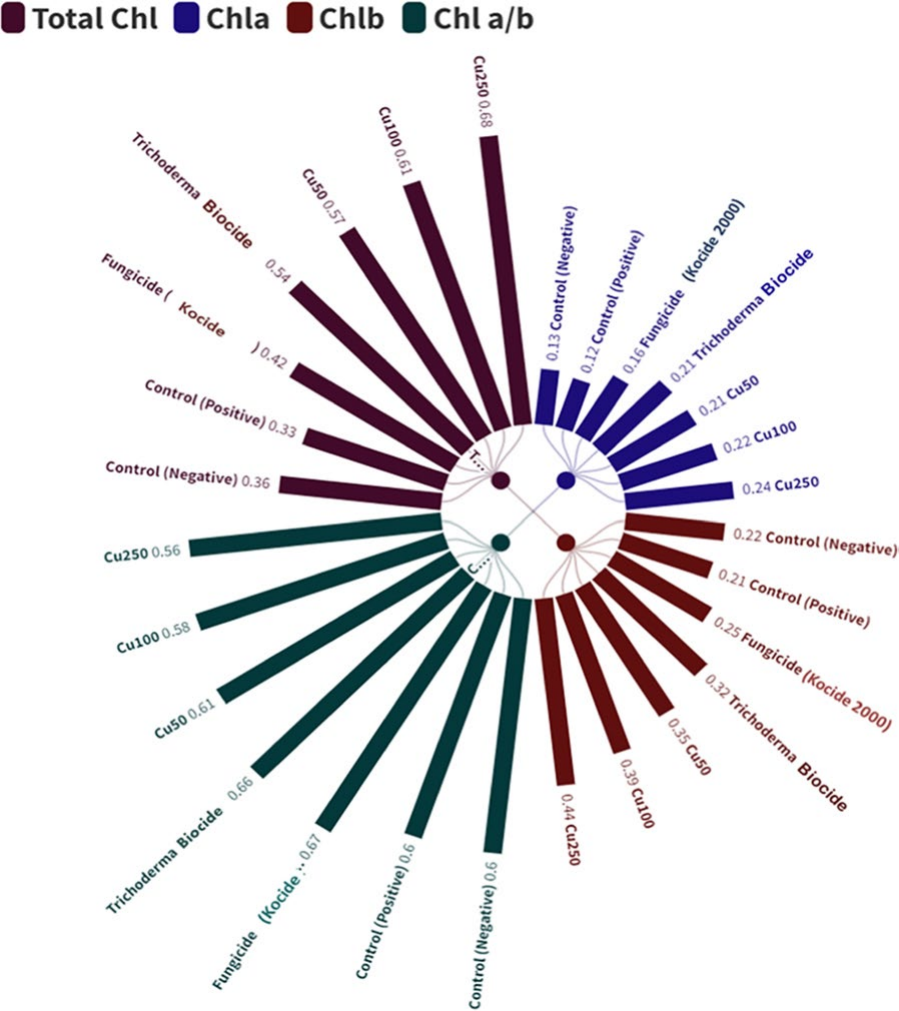
Effect of different concentrations of CuO-Zs-NPs (50, 100, 250 mg/L), the chemical fungicide "Kocide 2000" and Trichoderma biocide on Chlorophyll a, b, total chlorophyll and Chl a/b ration of tomato plants treated with *F. solani*

To investigate the effect of different concentrations of CuO-Zs-NPs on chlorophyll concentration and composition in tomato plant leaves. The total chlorophyll concentration in the control of tomato plant leaves was 0.36 ± 0.014 mg /g fresh weight, whereas, in CuO-Zs-NPs (250.100 and 50, respectively), it was 0.68, 0.61, and 0.57. However, the composition of chl was significantly different, as evidenced by a decrease in the chl a/b ratio in CuO-Zs-NPs 250 (0.56), this ratio was increased when decreasing the concentration of CuO-Zs-NPs treatment compared to the negative control (0.60) (Fig. 10). While the chl a/b ratio increased in both the biotreatment and the Cu fungicide treatment to 0.66 and 0.67, respectively, and both the control and CuO-Zs-NPs 50 had similar cha/chb values.

The enhanced photosystem activity observed in plants exposed to nanoparticles (NPs) may be attributed to the up-regulation of genes related to photosynthesis, such as psaA (photosystem, I P700chlorophyll an apoprotein A1), pet A (photosynthetic electron transfer A), HSP90.1 (heat chock protein1), and psbA (photosystem, II reaction center protein A) [84]. Additionally, NPs may form complexes with light-harvesting complex (LHC) proteins in the antennae of photosystems, thereby improving light absorption [85]. Furthermore, NPs can increase CO_2_ assimilation by enhancing the activity of beta-carbonic anhydrase (BCA) and Rubisco enzymes in light-independent reactions [42, 43]. The promotion of photosynthetic activity by NPs is also responsible for the increased fresh and dry biomass of tomato plants compared to chemical fungicides or Trichoderma biocides. This effect is due to NPs increasing light absorption, accelerating energy transport between photosystems, promoting photolysis of water, and facilitating oxygen evolution [43]. Moreover, the results revealed a significant increase in chlorophyll a (ch-a), b (ch-b), and total chlorophyll content in tomato plants treated with CuO-Zs-NPs compared to other treatments. The concentration of CuO-Zs-NPs had significant effects on chlorophyll content (Fig. 10), with CuO100 and 250 inducing an increase of approximately 50% in chlorophyll a and about 40% in chlorophyll b compared to the control. Similarly, CuO50 also resulted in a moderate increase in chlorophyll a and b, albeit with a relatively smaller amount. All treatments showed an increase in chlorophyll b content, but tomato plants treated with either CuO50, CuO100, or CuO250 showed significant increases compared to those treated with Kocide 2000 chemical fungicide, biocide, or samples of untreated control. The stimulatory influence of CuO-Zs-NPs on the production of these pigments may be attributed to their ability to enhance plant nutrition by promoting nitrogen absorption. This process can significantly increase the concentration of nutrients in plants, leading to improved growth and increasing phenolic compound concentrations that enhance pigment production [82]. Additionally, the second stage of the bio-stimulation technique for CuO nanoparticles involves the bio-transformation of the core material into ions inside the cytoplasm. Specifically, this process converts CuO nanoparticles into Cu ions. This transformation is likely associated with an increase in chlorophyll levels, which suggests that the ions may be involved in promoting plant growth and photosynthesis. Overall, this second stage of bio-stimulation appears to be an important step in enhancing the effectiveness of CuO nanoparticles for agricultural applications [38]. The increase in chlorophyll a and b concentrations may be explained by the fact that treatments including varied doses of CuO50, CuO100, and CuO250 stimulated strong root development. Where treatments can improve root vitality as in maize, allowing the crop to absorb nutrients from the growing medium [55]. In addition to improving the crop's capacity to absorb light via its photosystems, due to increases in chlorophyll concentrations are thought to be a natural reaction to environmental stimuli [65].

The ratio of chlorophyll a/b is often used as a criterion to estimate crop responses to environmental stresses [78]. Some stresses can cause the conversion of chlorophyll a to b via the enzyme Chl-a oxygenase [13]. The decrease in the Chla/Chlb ratio observed in this study may be due to CuO-Zs-NPs promoting the activity of this enzyme [94]. This results in an increase in Chlb concentration and a reduction in the ratio, indicating a higher concentration of PSII against PSI. This finding is supported by previous research showing that Chlb is abundant in PSII [11, 81]. Increased PSII levels in leaves indicate greater efficiency in absorbing solar energy. Chl-b also plays an important role in arranging thylakoid membranes and regulating LHCs [90]. These results corroborate the earlier descriptions of plant dry matter increase and photosynthetic efficiency.

In conclusion, CuO-Zs-NPs have been found to promote strong root development and increase chlorophyll concentrations in tomato plants. The decrease in the Chla/Chlb ratio observed may be due to the increased activity of the enzyme responsible for synthesizing Chlb from Chla. These findings suggest that CuO-Zs-NPs may have potential applications for improving crop growth and productivity.

#### Effect of CuO-Zs-NPs on enzymatic activities of treated plants

**Fig. 11:**
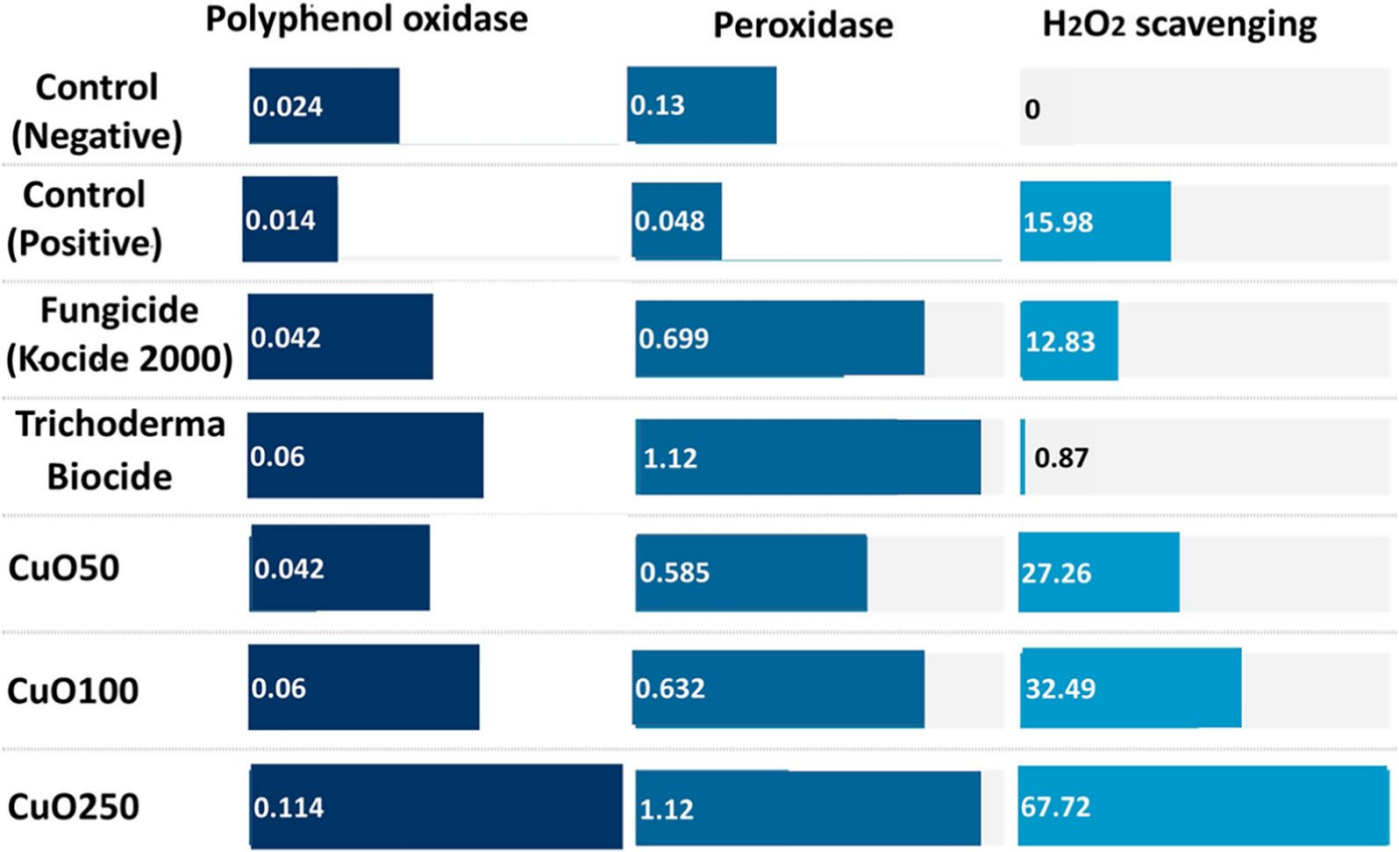
Effect of CuO-Zs-NPs 50, CuO-Zs-NPs 100, and CuO-Zs-NPs 250, the chemical fungicide “Kocide 2000” and Trichoderma biocide on some enzymatic activity "polyphenol oxidase, Peroxidase, H_2_O_2_ Scavenging" of tomato plants challenged with *F. solani*

In the pot experiment, to understand more about the interaction of *F. solani* with tomato plants following the application of CuO-Zs-NPs at varied concentrations. A number of defense-related enzymes were examined. The data obtained showed that the inoculation of *F. solani* improved the activity of all the defense-related enzymes examined in all treatments compared to the un-inoculated control plants. The CuO-Zs-NPs significantly modified the enzymatic activity of polyphenol oxidase and peroxidase in tomato leaves (Fig. 11). The interaction of CuO-Zs-NPs at 50, 100, and 250 mg/l increased the activity of the polyphenol oxidase enzyme to 75, 150 and 375% respectively compared to the non-treated healthy and infected tomato plants showing polyphenol oxidase enzymatic activity of 0.024 and 0.014 U/min/gram respectively. In contrast, treatment with the chemical fungicide and Trichoderma Biocide that increased polyphenol oxidase activity to 75, and 150% respectively.

On the other hand, the peroxidase enzyme was increased to 350, 386, and 762% respectively in comparison with the non-treated healthy and infected tomato plants that showed peroxidase enzymatic activity 0.13, 0.048 U/min/gram respectively. In contrast, treatment with the chemical fungicide and Trichoderma Biocide increased peroxidase activity to 415, and 762% respectively. Additionally, the activity of hydrogen peroxide (H_2_O_2_) scavengers as an antioxidant response to oxidative stress potentially caused by CuO-Zs-NPs treatments was determined in tomato plant leaves. The results indicated that both CuO-Zs-NPs and the chemical fungicide treatments exhibited higher levels of H_2_O_2_ scavenger activity than the Trichoderma biocide treatment and the untreated control (Fig. 11).

Overall, the results of our study indicate that the application of CuO-Zs-NPs exogenously can enhance the resistance of tomato plants against *F. solani* infection. This finding is consistent with previous reports demonstrating that certain derived compounds, when applied externally, can induce resistance in host plants by elevating the levels of host defense enzymes and pathogen-related (PR) proteins [36]. It is suggested that CuO-Zs-NPs may trigger systemic acquired resistance (SAR) in host plants, thereby reducing their susceptibility to infection, as described previously [36]. SAR is a plant immune response that prevents the spread of infection or disease to non-infected parts of the host plant and is typically characterized by an increase in PR proteins such as chitinases, -1,3-glucanases, acid invertases, and peroxidases [4, 10, 77]. Several studies have shown that substances derived from plants can stimulate the development of more resistant crops and reduce disease incidence [61, 67]. The induction of systemic resistance in plants involves the activation of dormant defense genes under various conditions.

Numerous studies have reported that increased levels of peroxidase, protease, and polyphenol oxidase (POD) enzyme activity, as well as the expression of genes for -1,3-glucanase and chitinase, are effective against various fungal diseases [61, 67]. This is in line with Fernandes, & Ghag, [25] provided an inclusive assessment of tomato genes involved in pathogen detection, defense signaling networks, and the functions of enzymes that support host resistance to *Fusarium solani*. CuO-Zs-NPs treatments at various concentrations have been shown to increase defense-related enzymes and pathogenesis-related proteins in tomato plants against root rot pathogens. These proteins may play a crucial role in strengthening the host plant's cell walls to resist *F. solani* infection. Additionally, CuO-Zs-NPs may activate defense mechanisms in response to pathogen inoculation by developing additional proteins to prevent pathogen entry or subsequent spread due to the importance of Cu as a microelement for plants. Furthermore, recent studies have also demonstrated the role of Cu Bio-nanoparticles in increasing H_2_O_2_ scavenger activity as an anti-resistance mechanism against stress or toxicity [87]. The application of Cu nanoparticles has been shown to enhance antioxidant enzyme activity and reduce oxidative damage caused by stress or toxicity. Therefore, it can be concluded that Cu nanoparticles have potential applications in enhancing plant defense mechanisms against various biotic and abiotic stresses.

### Determination of Cu content in terminal apical tomato tissues

Tomato crop growth is dependent on a range of essential nutrients, both macronutrients (such as N, P, K, Ca, and Mg) and micronutrients (such as Cu, Fe, B, Mn, and Mo). These nutrients are crucial for various processes including photosynthesis, cellular respiration, and defense responses. The roots are the primary site for nutrient absorption. Cu is particularly important in mitigating Reactive Oxygen Species (ROS), facilitating photosynthesis, regulating phenol metabolism, and promoting protein synthesis [86, 87]. As shown in Table 2, tomato plants treated with the chemical fungicide Kocide 2000, showed the highest conc. of Cu with a 17.244 ± 0.22 value, followed by 12.372 ± 0.22, 12.516 ± 1.2, and 15.344 ± 0.32 for CuO-Zs-NPs 50, 100, and 250 treatments respectively, compared to 10.983 ± 0.40 for treatment with Trichoderma biocide and 10.272 ± 0.24 and 10.297 ± 0.35 for negative and positive controls respectively. In view of the obtained results and inconsistent with other research [34], increasing the conc. of Cu particularly at their nanoscale could promote the growth of tomato roots which directly improved seed germination, plant height, and fresh and dry weight as indicated above in (Figs. 5, 8) without any adverse effects. This is in agreement with [54] who reported that exposure of plant leaves to varying concentrations of Cu NPs did not result in an increase in Cu content in the fruits. However, some studies have suggested that the accumulation of Cu, Fe, Mn, and Zn in leaves may occur through uptake by plants via leaf apertures such as stomata, trichomes, and hydathodes when applied in foliar or through water uptake via root absorption [6]. Micronutrient regulation, particularly Cu, occurs during different pathways of transport that facilitate migration to the needed areas. Ghasemi et al. [23] found that some nutrients may cause fluctuations in other microelements, which can cause them to accumulate in different tissues.

**Table 2:** The Cu content in tomato terminal bud tissues after applying the CuO NPs treatments in compared to treatment with the chemical fungicide,Trichoderma biocide and the (positive, negative) controls

Treatment	Cu conc.
	(mg/l)
CuO50	12.372 ± 0.22^b^
CuO100	12.516 ± 1.2^b^
CuO250	15.344 ± 0.32^c^
Trichoderma biocide	10.983 ± 0.40^a^
Fungicide (Kocide 2000)	17.244 ± 0.22^d^
Post. Control	10.297 ± 0.35^a^
Neg. control	10.272 ± 0.24^a^

Bars with the same letters are not statistically significant based on the least significant difference (LSD) test (*P* < 0.05)

This may explain the accumulation of copper (Cu) in tomato plant leaves when exposed to the fungicide Kocide 2000 at a high concentration of 2.5 g/L, which may not give the best positive effects on some growth and physiological parameters in compared to lower dosages of CuO treatments at the nanoscale as indicated above. These findings suggest that CuO-Zs-NPs nanoparticles can enhance root growth and nutrient absorption, which could be considered another reason for promoting tomato growth in compared to other treatments. Additionally, the use of CuO nanoparticles in soil may mitigate environmental contamination caused by excessive agrochemical use.

### Pollen grain analysis

**Fig. 12:**
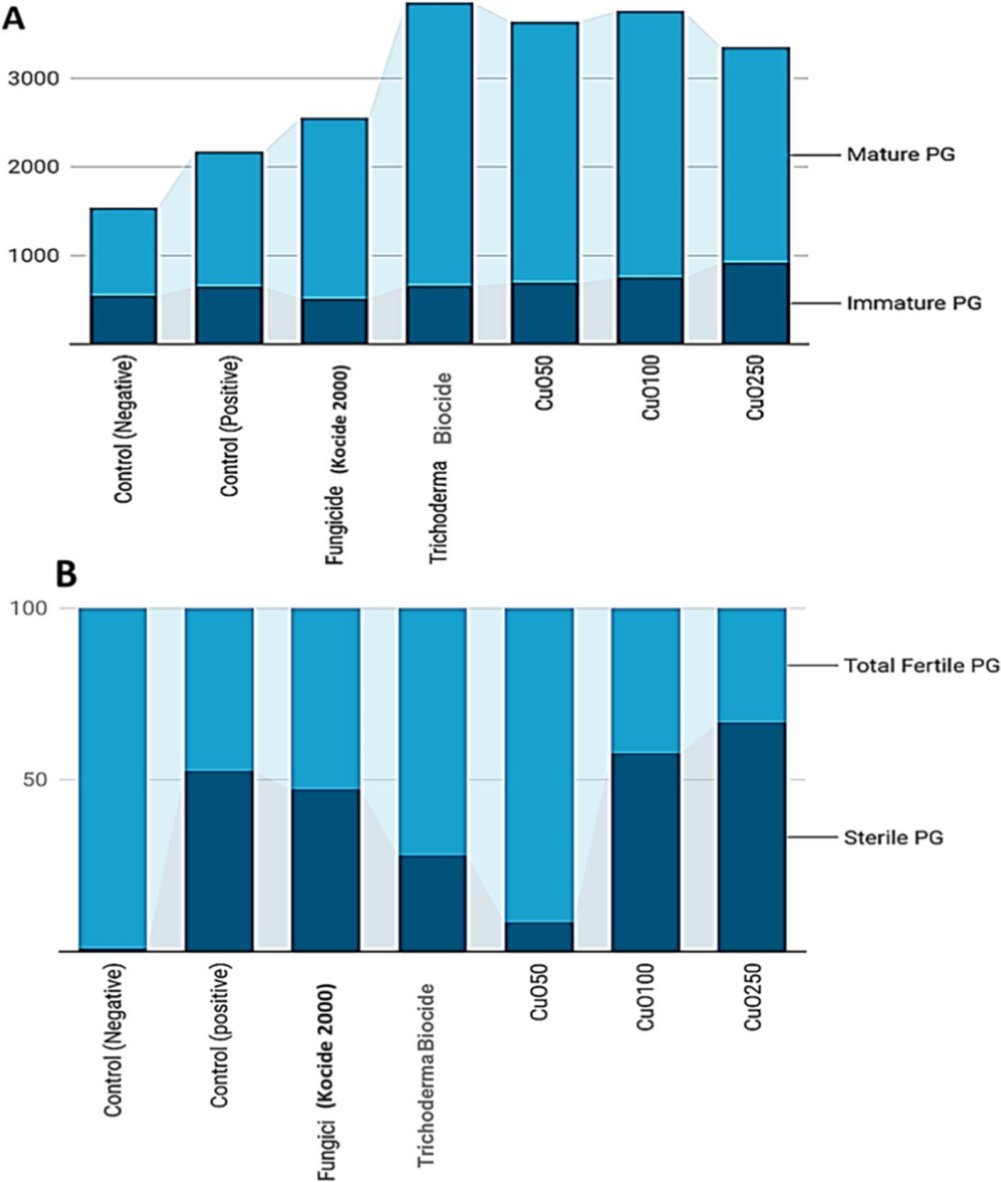
Effect of different concentration of CuO-Zs-NPs on the percentage ( %) of pollen grains maturity (**A**); and fertility in compared to treatment with the chemical fungicide and Trichoderma biocide (**B**)

**Fig. 13:**
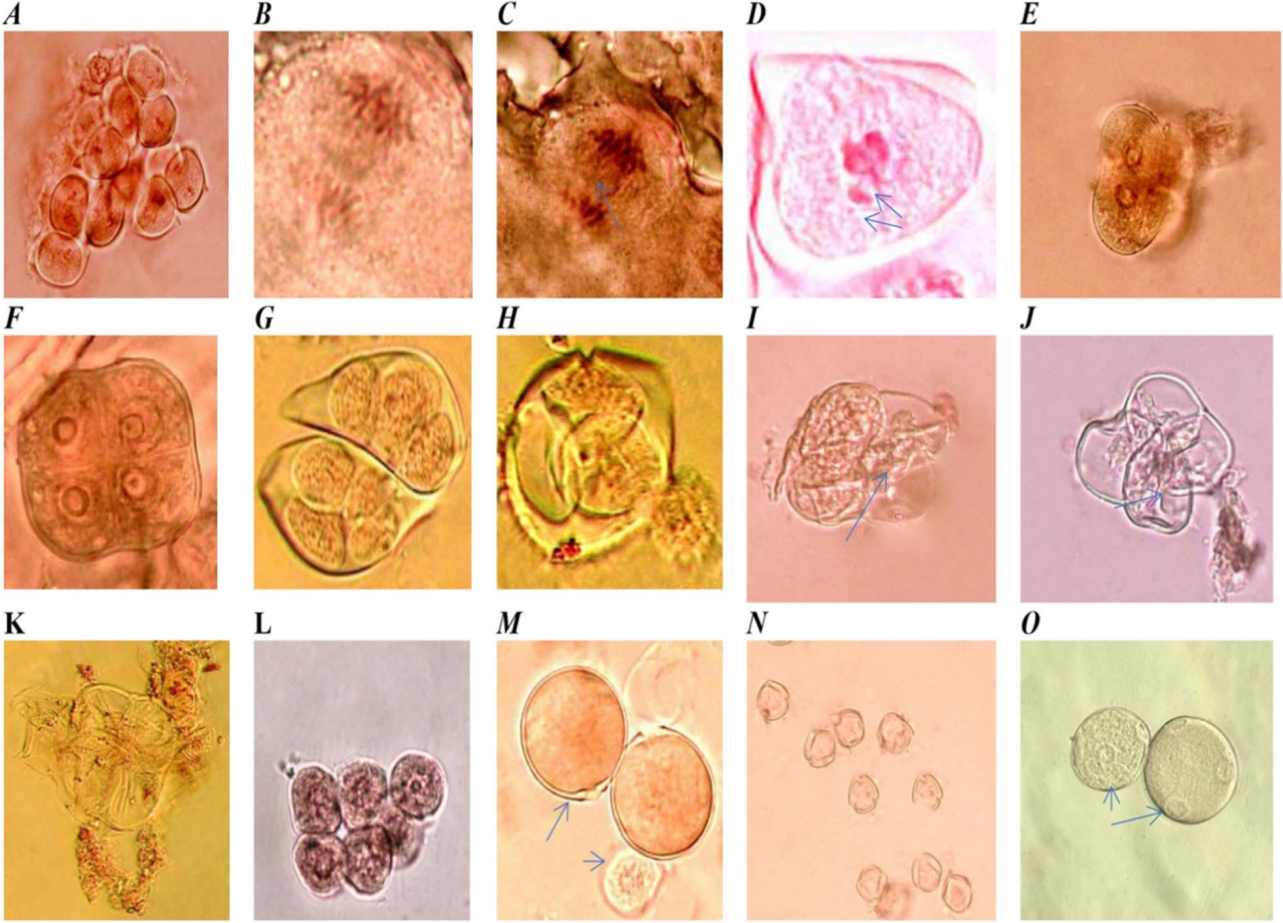
**A**. Divided cell in meiosis I and II; **B**. Normal anaphase I; **C**. Anaphase I with lagging chromosome arrow; **D**. Metaphase I with lagging chromosome and fragment arrow **E**. Diad pollen grain arrangements; **F**. Normal Microspore tetrad (isobilateral); **G**. Normal Microspore tetrad (Liner and Decussate) **H**. Tetrad release immature PG arrowhead (Tetrahedral); **I**. Two cells from tetrad lose cytoplasmic granules arrows; **J**. All cells formed tetrad lose cytoplasmic granules; **K**. cytoplasmic granules outside tetrad arrows; **L**. immature PGs; **M**. Mature Fertile PGs (reduce) arrow and immature arrowhead; **N**. Sterile PGs; **O**. Mature fertile 2n pollen grain arrow (unreduced)

The data in Table (3) indicate that the effect of Cu nanoparticles on the pollen grain fertility of tomato plants was analyzed. The results indicated that treatments with CuO-Zs-NPs at the lowest concentrations led to a rising number of mature pollen grains (Fig. 13M) compared to the immature ones (Figs. 12A and 13L). This is possibly, due to the early flowering of the plants treated with CuO-Zs-NPs. Which can affect the photosynthesis and respiration process of the plants, and influence the production of sugars and energy that are required for flowering. Similarly, treatment with Trichoderma (biocide) showed a noticeable increase in the number of mature pollen grains compared to the control samples. These findings are in agreement with data obtained by Marmiroli et al. [56], who reported that no aspect of the plant's appearance, development, or pollen formation changed after being treated with high concentrations (320 mg/kg^−1^) of nano-CuO. in contrast, some reports revealed that copper NPs decreased black cumin's fertility, suggesting that these NPs may have acted as a barrier to pollen formation or to the plant's normal development and maturation [48]. Additionally, fertile pollen grains of abnormal size in diploid 2n (Fig. 13O arrow) were observed in compared to normal (n) pollen grains (Fig. 13M), where meiosis was perfectly normal in the control treatment (Fig. 13A, B and E). Fertile n was found to be elevated to 91.5 in CuO-Zs-NPs at a concentration of 50mg/l, approaching the values (99 ± 8.7 of untreated (negative control, and there was no significant difference between them. On the other hand, the obtained results indicated that exposure of tomato tissues to Cu-Zs-NPs, particularly at the highest conc. (250 mg/l inhibited pollen development in the flowering stage. This is also suggested to have an adverse effect on pollen fertility which was recorded (30.1 ± 1.7% for n fertile pollens and (3.1 ± 0.2for 2n fertile pollens; subsequently, the total fertile pollens for this treatment were recorded (33.22% (Table 3).

**Table 3:** Effect of CuO-Zs-NPs treatments at thethree different concentrations(50, 100, and 250) mg/l on the percentage (%) of pollen grain fertility in compared to the chemical fungicide and Trichoderma biocide treatments

Treatments	N of Tetrad	N of Immature PG	Total N mature PG	Sterile PG %*	Fertile PG		
					n%*	2n%*	Total fertile
CuO50	70	689	2950	8.5 ± 0.2^E^	91.5 ± 0.3^A^	0 ± 0^C^	91.53
CuO100	75	750	3008	57.7 ± 2.2^B^	39.8 ± 2.0^CD^	2.4 ± 0.1^B^	42.31
CuO250	110	915	2435	66.8 ± 5.6^A^	30.1 ± 1.7^D^	3.1 ± 0.2^B^	33.22
Trichoderma biocide	50	655	3200	28.1 ± 2.8^D^	68.7 ± 2.9^B^	3.1 ± 0.2^B^	71.88
Fungicide	150	514	2040	47.3 ± 1.6^C^	49.7 ± 4.1^C^	2.9 ± 0.1^B^	52.7
Positive Control	100	650	1520	52.6 ± 0.7^BC^	35.6 ± 0.1^CD^	11.8 ± 0.4^A^	47.43
Untreated Control (Negative)	212	544	1000	1 ± 0.2^E^	99 ± 8.7^A^	0 ± 0^C^	99

*N:* number.n, haploid. 2n diploid*% percentage from total mature PG; Bars with the same letters are not statistically significant based on the least significant difference (LSD) test (*P* < 0.05)

While the treatment with biocide recorded (68.7 ± 2.9) for the haploid (n) fertile pollen grain and (3.1 ± 0.2) for the diploid pollen, resulting in a total of 71.88% fertile pollen (Fig. 12B). Then, the chemical fungicide was recorded (49.7 ± 4.1) in haploid pollens and (2.9 ± 0.1) in diploid pollens to enhance the number of total fertile pollens to 52.7%. Additionally, there were slight increases in the 2n% value and no significant difference among the treatments with CuO-Zs-NPs at conc. 100 mg/l (2.4 ± 0.1), the chemical fungicide (2.9 ± 0.1), CuO-Zs-NPs at conc. 250 mg/l (3.1 ± 0.2), and Trichoderma biocide (3.1 ± 0.2). Treatment with *F. solani* only increased the diploid 2n to 11.8%. To explain this, we suggest that the damage caused by *F. solani* to tomato root tissues leads to a functional impairment that occurs from nutritional imbalances, which had the greatest effect on the abnormal appearance [49]. On the other hand, other reports suggest that the effect of CuONPs possibly prevents centromeric division, leading to the production of extra chromosomes (Diplo-chromosomes) [48]. Additionally, polyploid cells and stragglers can be caused by defects in the spindle process.

ANOVA statistical analyses indicated a noticeable significant difference (P < 0.05) between most of the experimental groups compared to the control group of sterile PG (Fig. 13N). However, when comparing the fungicide-positive control with the copper-based Cu 100, there was no statistically significant difference (P > 0.05) (Table 3). The results indicated a noticeable sterile pollens (unstained pollens, Fig. 13N) which increased significantly in the treatment with CuO-Zs-NPs at conc. 250 mg/l, which was 66.8 ± 5.6, but there was no significant difference between the treatment with CuO-Zs-NPs at conc. 100 mg/l, 57.7 ± 2.2, and the positive control, 52.6 ± 0.7. On the other hand, the biocide treatment recorded a moderate rate (28.1 ± 2.8). The results also showed no significant difference between the positive control and the treatment with the chemical fungicide (47.3 ± 1.6). While the best treatment was CuO-Zs-NPs at a concentration of 50 mg/l giving (8.5 ± 0.2), where there was no noticeable significant difference with the negative control (1 ± 0.2).

Although treatments with CuO-Zs-NPs (100, 250 mg/l), and the chemical fungicide decreased the passive effect of *F. solani* on the tomato host plant, the treatments increased the negative effect (sterility) on pollen grains resulting in chromosomal lagging and fragments that induced mutations (Fig. 13C, and D). The decrease in the proportion of viable pollen grains in these treated plants relative to untreated plants can be attributable to their pollen-toxic effects. In fact, this harmful effect becomes more apparent when fungicides and high concentrations of CuO-Zs-NPs are used. Sterility occurs during the formation of gametophytes, most likely initiating at the microspore stage, and gametophytes are aborted at the beginning of their development. These findings are contrary to data obtained by Marmiroli et al. [56], who reported no changes in the development or viability of pollen after treatment with high concentrations (320 mg/kg) of nano-CuO.

The diploid gametes (Fig. 13O) may result from a variety of cytological abnormalities due to the five main cytological mechanisms of 2n gamete formation: pre-meiotic chromosome doubling during the transition from mitosis to meiosis, meiosis disturbances, and abnormal cytokinesis during the first or second meiosis resulting in dyad and triad formation (Fig. 13E, H), followed by 2n pollen formation. In this regard, some studies estimated the frequency of 2n pollen grains and suggested that they came from restoration nuclei generated during meiosis I and II as dyads and triads in the spore stage (Xue, Liu, and Liu [92]). Large-sized 2n pollen grains were observed to be well-filled, pigmented, and viable; hence, it is highly probable that fertilization by these 2n gametes can result in intraspecific polyploidy. Additionally, Sabrine et al., [74] mentioned that higher Cu concentrations disrupt critical functions such as mitosis and photosynthesis, causing plant tissue toxicity [70, 74]. Furthermore, heavy metals disrupt the mitotic cycle, cause chromosomal aberrations microtubule damage irregularly shaped nuclei, and decompose nuclear material [91], Eun, Shik Youn, and Lee [22]).

The results recorded a clear observation in the frequency pattern of a normal tetrad (isobilateral) and tetrads liner and Decussate (Fig. 13F, G) in comparison to that in the case of the frequency pattern of tetrad release immature PG (Tetrahedral), which showed that treatment with CuO-Zs NPs results in a clear observed loss of cytoplasmic granules in tetrad cells either outside or inside cells (Fig. 13H–K), such as the non-disjunction of chromosomes in both meiotic divisions. These findings can be explained by manipulations in male meiosis as indicated in Berdnikov et al [9]. Additionally, the treatments induce structural damage to the cell in the tetrad-stage pollen cytoplasm Grain (PCG) are naturally released from the pollen grain when the cytoplasm is expelled from the pollen grains through the pore. However, release could also occur through cracks of the exine when the pollen was damaged. Only a small proportion of the pollen releases their cytoplasm, and the remaining pollen remains intact. However, in fragile pollen, PCG release can also occur through breaks of the exine cytoplasmic granules that can be seen next to the pollens that come out of them. Our results showed that cytoplasmic granules were expelled from the cells in tetrad stages. Tetrad cytoplasmic granules can be seen next to tetrad stage also. Furthermore, the tetrads with two deadly pollen grains may result primarily from non-disjunction in anaphase I, and those with one pollen grain may result primarily from non-disjunction in anaphase II [9]. This phenomenon results from the interaction of cell division and pollen development with some toxic treatments and increases the proportion of cytoplasmic granules released. These reasons also decreased the final percentage of total fertile pollen grains in the treated plants compared with the control, which can be attributed to their toxic effect on pollen.

## Conclusions

The present study explores the usage of *Ziziphus spina-Christi* wild leaf extract in green chemistry to synthesize copper oxide nanoparticles (CuO-Zs-NPs) for combating *Fusarium solani* that causes root rot on tomato plants. The results indicated that CuO-Zs-NPs exhibited superior activity in both laboratory and greenhouse experiments against Fusarium root rot more effectively than both commercial fungicides “Kocide 2000” and Biocide(*Trichoderma viride* 1.5% W.P) under in vitro and in vivo conditions. Interestingly, Cu concentration was higher in tomato plants treated with chemical fungicides compared to CuO-Zs-NPs, but at their nano scale, they significantly enhanced nutrient absorption,  root growth, improving seed germination, plant height, fresh and dry weight, photosynthetic and enzymatic parameters. Treatment with Trichoderma Biocide and CuO-Zs-NPs at low concentrations led to a rise in mature pollen grains, but at high concentrations, it increased the percentage of sterile PG and induced Tetrad cytoplasmic granules. The unique antifungal properties of CuO-Zs-NPs make them a promising bio-nanomaterial for crop protection strategies and could replace synthetic fungicides as safer alternatives. However, further research is still needed to fully understand the structure -activity relationship of CuO-Zs-NPs, scale up their synthesis procedure, and produce stable formulations with desirable characteristics.

## Data Availability

The datasets generated and/or analyzed during the current study are available in within the article.
