# Correction: Antifungal activity of copper oxide nanoparticles derived from *Zizyphus spina* leaf extract against Fusarium root rot disease in tomato plants

**DOI:** 10.1186/s12951-024-02307-9

**Published:** 2024-02-16

**Authors:** Sozan E. El-Abeid, Mohamed A. Mosa, Mohamed A. M. El-Tabakh, Ahmed M. Saleh, Mohamed A. El-Khateeb, Maha S. A. Haridy

**Affiliations:** 1 Nanotechnology & Advanced Nano-Materials Laboratory (NANML), Plant Pathology Research Institute, Agricultural Research Center, Giza, 12619 Egypt; 2 Mycology and Disease Survey Research Department, Plant Pathology Research Institute, Agricultural Research Center, Giza, 12619 Egypt; 3 Zoology Department, Faculty of Science, Al-Azhar University, Cairo, Egypt; 4 Department of Pharmaceutical Chemistry, Faculty of Pharmacy, Horus University, Horus, 34518 Egypt; 5 Water Pollution Research Dep, National Research Centre, Dokki, Egypt; 6 Central Lab of Organic Agriculture, Agricultural Research Center (ARC), 9 Gamaa St, Giza, 12619 Egypt

**Correction: Journal of Nanobiotechnology (2024) 22:28**

10.1186/s12951-023-02281-8

Following publication of the original article [1], it was noted due to a typesetting error the figures and tables were positioned incorrectly in the PDF version of the article.

**Figure 1**:

The incorrect position is: Page 7

The correct position is: Page 8 " after title: Green synthesis of CuO Zs NPs”

**Figure 2**:

The incorrect position is: Page 8

The correct position is: Page 9 “after title: Morphology and size distribution of CuO NPs”

**Figure 3**:

The incorrect position is: Page 9

The correct position is: Page 10 " after title: FT IR analysis”

**Figure 4**:

The incorrect position is: Page 10

The correct position is: Page 12 “after title: In vitro antifungal activity effect of CuO Zs NPs”

**Figure 5**:

The incorrect position is: Page 11

The correct position is: Page 13 “after title: Effect of CuO Zs NPs treatments on seed germinationand growth vigor”

**Figure 6**:

The incorrect position is: Page 12

The correct position is: Page 13 “after title: Effect of CuO Zs NPs treatments on seed germinationand growth vigor”

**Figure 7**:

The incorrect position is: Page 12

The correct position is: Page 14 after title: Effectiveness of CuO Zs NPs against Fusarium root rot disease infecting tomato plants”

**Figure 8**:

The incorrect position is: Page 13

The correct position is: Page 15 “after title: Growth and Physiological parameters Effect on plant height, weight, and chlorophyll content”

**Figure 9**:

The incorrect position is: Page 14

The correct position is: Page 16 “after title: Growth and Physiological parameters Effect on plant height, weight, and chlorophyll content”

**Figure 10**:

The incorrect position is: Page 14

The correct position is: Page 16 “after title: Growth and Physiological parameters Effect on plant height, weight, and chlorophyll content”

**Figure 11**:

The incorrect position is: Page 16

The correct position is: Page 17 “after title: Effect of CuO Zs NPs on enzymatic activities of treated plants”

**Figure 12**:

The incorrect position is: Page 18

The correct position is: Page 20 “after title: Pollen grain analysis”

**Figure 13**:

The incorrect position is: Page 19

The correct position is: Page 21 “after title: Pollen grain analysis”

**Table 1**:

The incorrect position is: Page 10

The correct position is: Page 12 “after title: In vitro antifungal activity effect of CuO Zs NPs”

**Table 2**:

The incorrect position is: Page 16

The correct position is: Page 17 “after title : Determination of Cu content in terminal apical tomato Tissues”

**Table 3**:

The incorrect position is: Page 19

The correct position is: Page 21 after title " Pollen grain analysis”

The original article [1] has been corrected.
